# Clinical measures in chronic neuropathic pain are related to the Kennedy and endocannabinoid pathways

**DOI:** 10.1111/eci.14351

**Published:** 2024-11-15

**Authors:** Stephanie L. Bourke, Eva Gonzalez Suarez, Barira Islam, John Stephenson, David P. Finn, Patrick C. McHugh

**Affiliations:** ^1^ Pharmacology & Therapeutics, School of Medicine, Galway Neuroscience Centre and Centre for Pain Research University of Galway Galway Ireland; ^2^ Centre for Biomarker Research School of Applied Sciences Huddersfield UK; ^3^ Department of Pharmacy School of Applied Sciences Huddersfield UK; ^4^ School of Human and Health Sciences University of Huddersfield Huddersfield UK

**Keywords:** 2‐arachidonoylglycerol, biomarker, choline phosphotransferase, clinical neuropathic pain, endocannabinoids, phosphatidylcholine

## Abstract

**Background:**

Chronic neuropathic pain (CNP) is a debilitating condition, often refractory to currently available drugs. Understanding biochemical alterations in peripheral tissues such as blood will be useful for understanding underlying pathophysiological processes relating to CNP.

**Methods:**

We collected blood from two independent cohorts of CNP and pain‐free controls (CNP *n* = 129/Controls *n* = 127) in the UK and Ireland to investigate the relationship between CNP‐associated molecular/biochemical alterations and a range of clinical and pain metric parameters. Multiple statistical comparisons were conducted on the data, with selected variables included in one or more of the intended inferential analyses (six models).

**Results:**

Gene expression analysis showed that choline phosphotransferase (*CHPT1*) was increased (*p* < .001) in the CNP group compared to controls. The levels of phosphatidylcholine, a metabolite of *CHPT1* in the Kennedy Pathway, were significantly (*p* = .008) decreased in the plasma of patients with CNP. Given the relationship between the Kennedy pathway and endocannabinoids, plasma endocannabinoids and related *N‐*acylethanolamines were quantified in clinical samples by HPLC‐Tandem Mass Spectrometry. Plasma levels of the endocannabinoid 2‐arachidonoylglycerol were higher in CNP samples compared to controls, and in the statistical models applied, 2‐arachidonoylglycerol significantly increased the odds of CNP (*p* < .001). The expression of genes related to the synthesis and catabolism of endocannabinoids also corroborated the increased plasma 2‐arachidonoylglycerol levels in patients with CNP.

**Conclusions:**

Endocannabinoid levels, expression of genes related to endocannabinoid metabolism, age, sex, depression and anxiety state together were strong predictors of CNP. The observed molecular changes indicate that lipid metabolism is altered in CNP and thus may represent a viable target for novel analgesics or biomarker development.

## Graphical abstract

We have observed alterations in components of the Kennedy pathway in CNP including upregulation of choline phosphotransferase (*CHPT1*) and a lower concentration of PC in the blood of patients with chronic neuropathic pain. Moreover, higher circulating levels of the endocannabinoid 2‐AG and downregulation of the 2‐AG hydrolysis enzyme *MGLL* were observed in patients with CNP. Endocannabinoid levels, expression of genes related to EC metabolism, age, sex, depression and anxiety state together were strong predictors of CNP.

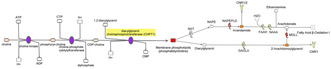

## 1 INTRODUCTION

Chronic neuropathic pain (CNP) is a debilitating condition affecting 7%–10% of the population worldwide.^1^, ^2^ CNP is caused by damage to, or dysfunction of, the peripheral or central divisions of the somatosensory system.^1^ CNP involves sensitisation of central nociceptive neurons to afferent input, which may persist for long periods (e.g. years).^3^ Drug management of CNP provides short‐term symptomatic relief in some patients but there are often undesirable side effects from these medications.^1^, ^4^ At present, there is a lack of objective biomarkers to guide diagnosis and choice of drug treatment for CNP,^5^ and understanding mechanisms relating to CNP may hold the key. A complete understanding of the mechanism(s) of CNP is complicated by multiple pain aetiologies and the involvement of many molecular and cellular pathways contributing to an individual's pain experience.^6^

Studies that have contributed to our understanding of the mechanisms underpinning CNP have utilised methods such as transcriptome analysis, protein expression techniques and animal models.^5^, ^7^, ^8^, ^9^, ^10^ Nevertheless, there is a need for comprehensive studies to contextualise how signalling pathways and metabolites synchronise in CNP. Notably, most of the studies in CNP biomarker and drug‐target discovery have been carried out in rat models, and cross‐species validation is a challenge due to fundamental metabolic differences between rodents and humans.^7^, ^10^, ^11^

In a previous study exploring molecular profiles in blood from patients with chronic pain, we identified candidate molecules that were significantly dysregulated in patients with CNP compared to controls, including the gene *CHPT1*.^9^ Further analysis using the Leeds Assessment of Neuropathic Symptoms and Signs (S‐LANSS) diagnostic questionnaire revealed higher expression of *CHPT1* in patients with CNP with an S‐LANSS score ≥12, an indicator of pain with a neuropathic origin.^12^

CHPT1 regulates the de novo biosynthesis of phosphatidylcholine (PC) in the last step of the Kennedy pathway through the intermediate cytidine 5′diphosphocholine (CDP‐choline), where CHPT1 catalyses phosphocholine to diacylglycerol to generate PC.^13^ PC is one of the main constituents of the cell membrane, providing structure and integrity to the cell. In mice, synergistic antinociceptive effects of choline and low doses of morphine have been demonstrated in the formalin test of inflammatory pain.^14^ Moreover, CDP‐choline administration has been shown to prevent the development of neuropathic pain‐related behaviour and promote nerve regeneration after sciatic nerve crush injury in rats.^15^, ^16^

Emerging evidence indicates that cells, upon tissue damage/injury, release lipid‐derived molecules that can modulate CNP.^17^ These include endocannabinoids (ECs) and oxidative products of long‐chain polyunsaturated fatty acids, which play a role in the peripheral response to nociceptive stimuli.^17^ ECs are synthesised from phospholipids, including PC.^18^ The EC system modulates pain, and evidence from preclinical studies utilising EC system modulators demonstrates the role of the EC system in CNP.^19^, ^20^, ^21^, ^22^, ^23^

For the study described herein, we aimed to confirm our previous finding with *CHPT1* expression levels and further explore levels of PC and components of the EC system in a combined independent cohort analysis in the blood of CNP and control participants.

## 2 METHODS

### 2.1 Study design and participants

Samples were collected across two independent sites, in the United Kingdom (cohort A, 113 samples in total) and Ireland (cohort B, 143 samples in total). Samples for cohort A, 51 CNP patients and 62 pain‐free healthy controls, were collected at the University of Huddersfield (control participants) and the Pain Management Services at Seacroft Hospital – Leeds Teaching Hospitals Trust, Leeds, United Kingdom and have previously been reported.^9^ Samples for cohort B, 78 CNP patients and 65 pain‐free healthy controls, were collected at the Health Research Board Clinical Research Facility, University of Galway, Ireland. The pain patients of both cohorts were recruited through the clinic based on CNP (>3 months) of any type as their clinical diagnosis. Patients were excluded for any current diagnosis of diabetes, cancer, osteoarthritis, fibromyalgia, major psychiatric conditions and other complex metabolic diseases which could alter their molecular profiles. Controls did not have any symptoms of CNP or any underlying conditions.

Data for age, sex, the Patient Health Questionnaire (PHQ‐9) and the state anxiety component of the State–Trait Anxiety Inventory (STAI) questionnaire, respectively, were collected (Tables S1 and S2). For the STAI, we focused on the state anxiety (STAI‐I) as this measure is more temporally relevant to our pain and endocannabinoid measurements. Clinical data related to pain, including pain duration in months, self‐reported pain, S‐LANSS, Chronic Pain Grade and current medications, were also recorded for CNP participants.

The blood samples used in this study were obtained with informed consent from the patients. Venous blood was collected from the antecubital fossa of all participants using standard phlebotomy technique, and RNA/plasma was extracted (Appendix S1 for more details on methods). Ethics was sought and approved through the Health Research Authority Research Ethics Committee (14/YH/0117) for cohort A and NUI Galway Clinical Research Ethics Committee (C.A. 1037) for cohort B. All the methods were performed in compliance with the institutional protocols.

### 2.2 Real‐time polymerase chain reaction (qRT‐PCR) analysis

The qRT‐PCR of selected genes (Table 1) was carried out with cDNA synthesised using whole blood total RNA (see Appendix S1) for a larger subset of participants in both cohorts. The relative gene expression of the markers was normalised to the geometric mean of *GAPDH, ACTB* and *SDHA* and then compared to the control group, according to the $2^{–\Delta \Delta C_{t}}$ method.^24^ Further details of the primers and qRT‐PCR conditions are presented in Appendix S1.

**TABLE 1:** Summary of genes and the function of the encoded proteins related to the EC system.

Gene	Protein encoded	Function	Ref
*CHPT1*	Choline Phosphotransferase 1	Catalyses the conversion of CDP‐choline and diacylglycerol to PC	^68^
*DAGLA*	Diacylglycerol lipase	Catalyses diacylglycerol to generate 2‐AG	^69^
*NAPEPLD*	*N*‐acylphosphatidylethanolamine Phospholipase D	Catalyses the release of *N*‐acylethanolamine	^70^
*MGLL*	Monoacylglycerol lipase	Hydrolyses 2‐AG	^71^
*FAAH*	Fatty acid amide hydrolase	Hydrolyses fatty acid amides such as AEA, PEA and OEA	^72^
*NAAA*	*N*‐acylethanolamine acid amidase	Hydrolyses AEA, PEA and OEA	^73^
*CNR1*	Cannabinoid receptor 1	Involved in the regulation of pain, mood, appetite, vascular and nonvascular smooth muscle tone and immune function	^74^

### 2.3 Estimation of PC in plasma samples

PC content in the plasma (see Appendix S1 for more details) was quantified using the colorimetric assay kit from Abcam, Cambridge, UK (ab83377). This assay uses an enzyme‐coupled reaction to hydrolyse PC and release choline, which subsequently oxidises 1‐(3,7‐Dihydroxyphenoxazin‐10‐yl) ethanone (OxiRed probe), resulting in the development of colour which could be quantified by reading the absorbance at 570 nm. The plasma samples were diluted to 1 in 200, and the assay was carried out in duplicate for each sample as per the manufacturer's instructions. The concentration of PC in the plasma samples was estimated using the standards supplied in the assay kit. The concentration of plasma proteins was calculated using the Bradford assay (#5000002) (Bio‐Rad Laboratories, Deeside, United Kingdom). A BSA standard curve was made (0, .0625, .125, .25, .5 and 1 g L^−1^) and absorbance was read at 595 nm. No significant differences were observed in the plasma protein concentration. Both the PC and protein estimations were carried out in duplicate.

### 2.4 Quantification of plasma endocannabinoids and related N‐acylethanolamines by HPLC – Tandem Mass Spectrometry

Plasma levels of ECs, 2‐AG and anandamide (AEA), and related *N*‐acylethanolamines, *N*‐oleoylethanolamine (OEA) and *N*‐palmitoylethanolamide (PEA), were quantified in 96% of the plasma samples (ECs could not be quantified in the remaining 4%) by HPLC coupled to Tandem Mass Spectrometry (LC–MS/MS). Plasma proteins were precipitated and removed from the plasma by centrifugation. The supernatant was filtered, dried and resuspended in acetonitrile (ACN). Mobile phases consisted of (A) HPLC‐grade water with .1% (v/v) formic acid and (B) ACN with .1% (v/v) formic acid with a flow rate of .2 mL/min using an Agilent ZORBAX RRHT C18 column (2.1 × 50mm, 1.8 μm, 80 Å, 600 bar pressure limit). Analyte detection was carried out in electrospray‐positive ionisation mode on an Agilent 1260 infinity 2 HPLC system coupled to a SCIEX QTRAP 4500 mass spectrometer operated in triple quadrupole mode (SCIEX Ltd., Phoenix House Lakeside Drive Centre Park, United Kingdom) (see Appendix S1 for further details). Mass spectrometry was performed at the University of Galway.

### 2.5 Statistical analysis

Statistical analyses were carried out using IBM SPSS Statistics version 26. The samples were summarised descriptively. Univariate analyses were performed assessing the levels of endocannabinoids and related *N*‐acylethanolamines, gene expression and questionnaire data in patients with CNP compared to controls. As PC was estimated on a smaller subset of samples, its levels in CNP versus control samples were compared using an independent samples *t*‐test. *p* < .05 was considered statistically significant.

Exploratory correlational analyses were conducted on outcome variables to identify highly correlated variables and to assess the suitability of data for a multivariate treatment. A further exploratory analysis was conducted to assess the extent of any systematic differences between the two sites. Missing gene expression data was estimated to be missing at random and possibly missing completely at random and was hence suitable for imputation. Multiple imputations (using 5 iterations) were conducted on the data, using all variables included in one or more of the intended inferential analyses.

As the direction of associations between measured quantities is elusive; six models were made based on the following analyses (Figure S1):

Analysis 1: a multivariate linear regression assessing the effect of CNP, age and sex on gene expression levels.

Analysis 2: a multivariate linear regression assessing the effect of CNP, age and sex on EC concentrations.

Analysis 3: a multivariate linear regression assessing the effect of CNP, age and sex on depression and anxiety; measured by the PHQ‐9 and STAI‐I instruments as described above.

Analysis 4: a multiple logistic regression assessing the effect of gene expression levels, EC concentrations, age, sex, depression and anxiety on CNP.

Analysis 5: a structural equation model testing concurrently the effect of gene expression levels, EC concentrations, age, and sex (acting as exogenous variables) on CNP (acting as an endogenous variable) and CNP (acting as an exogenous variable) on depression and anxiety (acting as endogenous variables). The first part of the model was modelled using a multiple logistic regression model; the second part was modelled using a multivariate linear regression model.

Analysis 6: a structural equation model testing concurrently the effect of gene expression levels, EC concentrations, age, and sex (acting as exogenous variables) on depression and anxiety (acting as endogenous variables) and depression and anxiety (acting as exogenous variables) on CNP (acting as an endogenous variable). The first part of the model was modelled using a multivariate linear regression model; the second part was modelled using a multiple logistic regression model.

In all of the above analyses, CNP, gene expression levels, EC concentrations, and participant depression and anxiety were conceptualised as the key predictor or outcome variables of interest, with age and sex as controlling variables (Figure S1). The reference category for sex was set as *male*. The Akaike information criterion (AIC) and the Bayes information criterion (BIC) were derived for each model to compare the tested models in terms of the degree of parsimony.

## 3 RESULTS

### 3.1 Characteristics of study participants

This study recruited 256 participants, 127 pain‐free controls and 129 patients with CNP patients from two independent sites (Table 2). Cohort A comprised of samples from the United Kingdom (113 samples in total) while cohort B comprised of samples from Ireland (143 samples in total). The comparison of clinical characteristics revealed some differences in the participants recruited at the two sites included in the study. The proportion of CNP patients was higher in cohort B (78 out of 143 patients; 54.5%) than in cohort A (51 out of 113 patients; 45·1%) (Tables S1 and S2). The participants in cohort B (mean age = 51·1 years; SD = 13·4 years) were older than participants in cohort A (mean age = 41·8 years; SD = 13·7 years). Mean values of gene expression levels in CNP patients were similar across the two sites. Therefore, we carried out a meta‐analysis and compared the gene expression and EC levels from the combined cohorts.

**TABLE 2:** Characteristics of control participants and patients with neuropathic pain (i.e. cohorts A and B combined). Variables are summarised as mean (standard deviation) unless otherwise indicated.

Variable	Control participants (*n* = 127)	Neuropathic pain patients (*n* = 129)
Sex^a^	47 (37.9%)	54 (44.3%)
Male	77 (61.2%)	68 (55.7%)
Female	(*n* = 124)	(*n* = 122)
Age (years)	43.9 (15.0) (*n* = 124)	49.7 (13.0) (*n* = 119)
PHQ‐9 severity classification (score)	1.73 (2.39) (*n* = 124)	10.75 (7.09) (*n* = 118)
Minimal (0–4)	108 (87.1%)	28 (23.7%)
Mild (5–9)	15 (12.1%)	25 (21.2%)
Moderate (10–14)	1 (.8%)	32 (27.1%)
Moderately sever (15–19)		16 (13.6%)
Severe (20–27)		17 (14.4%)
STAI‐I score	28.6 (8.29) (*n* = 121)	42.9 (12.5) (*n* = 118)
Pain duration (months)	Non applicable	86.1 (89.1) (*n* = 128)
S‐LANSS score	Non applicable	13.2 (7.60) (*n* = 122)
S‐LANSS ≤11		48 (39.3%)
S‐LANSS ≥12		74 (60.7%)
Chronic pain grade	Non applicable	3.06 (1.00) (*n* = 118)
Anti‐inflammatory drugs taken^a^	Non applicable	
None taken		59 (55.1%)
Drugs taken		48 (44.9%) (*n* = 107)
Anti‐depression drugs taken^a^	Non applicable	
None taken		77 (72.0%)
Drugs taken		30 (28.0%) (*n* = 107)
Anti‐convulsant drugs taken^a^	Non applicable	
None taken		58 (54.2%)
Drugs taken		49 (45.8%) (*n* = 107)
Opioid drugs taken^a^	Non applicable	
None taken		55 (51.4%)
Drugs taken		52 (48.6%) (*n* = 107)
Plasma concentrations (pmol/mL)	3.37 (2.47)	8.1 (6.9)
2‐AG	1.26 (.76)	1.3 (.7)
AEA	9.9 (3.64)	10.4 (3.9)
PEA	7.88 (3.89)	8.5 (4.3)
OEA	(*n* = 119)	(*n* = 126)

Abbreviations: 2‐AG, 2‐arachidonoylglycerol; AEA, anandamide; OEA, N‐oleoylethanolamide; PEA, N‐palmitoylethanolamide.

^a^ Frequency (valid %).

Depression (PHQ‐9) and anxiety (STAI‐I) scores were significantly higher in patients with CNP compared to healthy controls (Table 2) (both *p* < .001). A score of less than 5 in the PHQ‐9 indicates the absence of a depressive disorder.^25^ In the present study, 76.3% of patients with CNP were classified as having mild to severe depression compared to 12.9% of healthy controls. At the time of the questionnaire, there was an average of 60.7% concordance with the diagnosis of neuropathic pain using the S‐LANSS across both cohorts.

Initial correlation analysis was carried out to determine whether the extent of correlation between variables would require the removal of one or more variables from further analyses. Only *DAGLA* and *FAAH* genes were strongly correlated (*r* = .755). No genes were removed from the analysis as a result of these findings. In the clinical characteristics, a high correlation was found between PHQ‐9 and STAI‐I scores (*r* = .746); however, the extent of the correlation between these variables was not sufficiently high to lead to singularities in variance–covariance matrices or to require the deletion of one of these variables.

The correlational analysis between the ECs revealed levels of AEA and related *N*‐acylethanolamines, PEA and OEA, to be highly mutually correlated (*r* = .866 or above). As AEA is one of the primary ECs, it was selected for further analysis alongside 2‐AG, which was not substantially correlated with AEA, PEA or OEA. The correlation between 2‐AG and AEA levels was .270; suggesting a multivariate treatment of these two variables would be appropriate. Fixed effect meta‐analyses of primary outcomes using weighted (unstandardised) mean differences revealed similar group effects across sites for 2‐AG, but moderate heterogeneity across sites for AEA (Figure S2). Overall, the evidence for heterogeneity across sites was inconclusive.

### 3.2 Expression of CHPT1 and PC are increased in CNP

**FIGURE 1:**
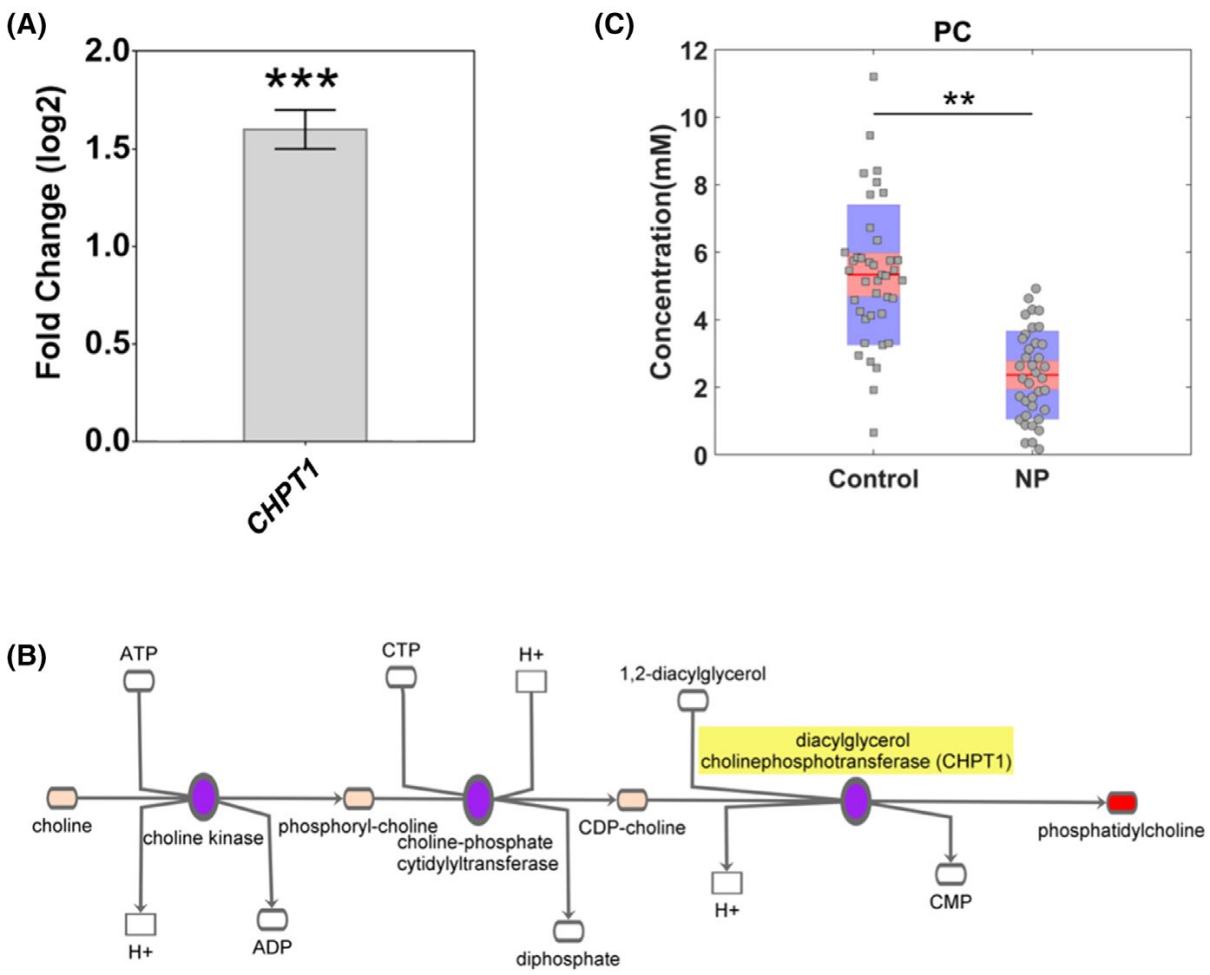
Expression analysis of *CHPT1* and levels of phosphatidylcholine (PC) in all clinical samples. (A) qRT‐PCR in 103 controls vs. 127 CNP samples showed statistically significant upregulation of *CHPT1* in CNP. The error bars show ±standard error of the mean (SEM). The *x*‐axis of bar graphs indicates the method of investigation. The fold change is shown in log2 scale and for qRT‐PCR, it was calculated by the $2^{-\Delta \Delta C_{T}}$ method. (B) Kennedy pathway showing the role of CHPT1 (highlighted in yellow) in the synthesis of PC (red box). (C) PC levels estimated in 40 control versus 37 CNP samples showed significantly lower levels in CNP (*p* = .008). In the box red line shows the mean and pink and blue area indicates values within a 95% confidence interval and standard deviation 1, respectively. The markers indicate the PC concentration in the samples. **p < .01; ***p < .001.

*CHPT1* was previously identified in Cohort A (UK) as significantly upregulated in CNP.^9^ To confirm this finding, we first looked to validate in cohort B (Ireland) alone with subsequent confirmation in a combined analysis of cohorts A and B by qRT‐PCR across all samples. Both analyses confirmed this previous *CHPT1* result where the gene expression was statistically significantly increased in CNP participants (Cohort B alone, *p* < .040; combined analysis, *p* < .001) with a 1.6‐fold upregulation in the combined cohort analysis (Figure 1A). Choline phosphotransferase 1, encoded by *CHPT1*, is involved in the synthesis of PC by the Kennedy pathway (Figure 1B).

Quantification of PC on randomly selected 40 control and 37 CNP samples and comparison by *t*‐test showed that PC levels were significantly lower in CNP compared to the controls (*p =* .008) (Figure 1C).

### 3.3 Plasma 2‐AG levels are increased in CNP

**FIGURE 2:**
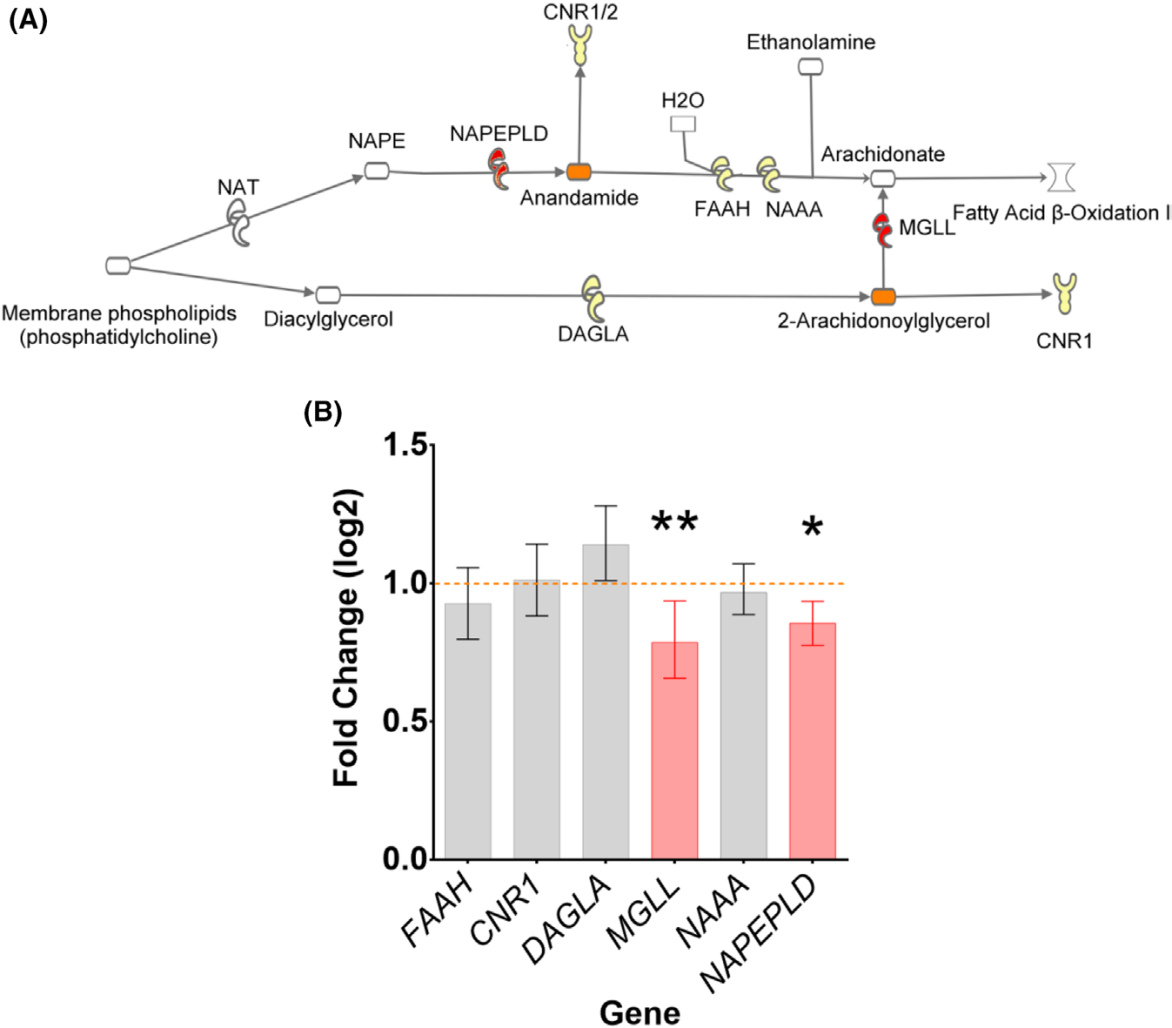
Investigation of EC pathway. (A) Anandamide (AEA) and 2‐arachidonoylglycerol (2‐AG) synthesis and hydrolysis routes with membrane phospholipids including PC as a substrate for synthesis. AEA and 2‐AG are shown in orange. The genes investigated by qRT‐PCR in cohort A are coloured; genes without significant difference are in yellow and genes with a significant change are in red. (B) qRT‐PCR of genes with samples from both cohorts combined showed significant downregulation of MGLL (*p* = .001) and NAPEPLD (*p* = .012) in CNP. Bar graphs of the genes significant to CNP/control group as a predictor are shown in red while non‐significant genes are shown in grey. The horizontal orange line marks the control at 1.00. *p < .05; **p < .01.

Upon discovering that PC levels were significantly lower in CNP compared to the controls, we searched the possible routes of PC utilisation within the body that may have a link to CNP. One of these pathways involves the production of ECs, 2‐AG and AEA from PC (Figure 2A). Therefore, we estimated the expression of genes involved in the EC pathway and compared using a multivariate model with age and sex as controlling variables (Analysis 1 in Table 3). The model revealed participant group (CNP or control) to be significantly related (at the 5% significance level) to a linear combination of outcome measures in an effect of low‐to‐medium magnitude (Wilk's Λ = .802; *F*_7246_ = 6.81; *p* < .001). The controlling variable of age was also significantly related to a linear combination of outcome measures (Wilk's Λ = .838; *F*_7246_ = 6.81; *p* < .001). Follow‐up univariate analyses revealed that significant differences between CNP or control group existed on *MGLL* with an effect of low magnitude (*F*_1252_ = 11.0, *p* = .001, partial‐η^2^ = .042) and *NAPEPLD* with an effect of low magnitude (*F*_1252_ = 6.39, *p* = .012, partial‐η^2^ = .035), but not on genes *FAAH*, *CNR1*, *DAGLA* and *NAAA* (Figure 2B and Table S3). Both *MGLL* and *NAPEPLD* were downregulated in the CNP group compared to controls (Figure 2B).

**TABLE 3:** Model parsimony statistics for the association of clinical characteristics, gene expression and EC levels.

Model	Predictors	Outcome	AIC	BIC	Fit
1	CNP, age, sex	Expression levels	32,159	32,345	Poor
2	CNP, age, sex	EC concentrations	−29,383	−29,330	Very good
3	CNP, age, sex	Depression & anxiety	20,510	20,563	Moderate
4	Gene expression levels, EC concentrations, age, sex, depression & anxiety	CNP	722.6	796.6	Good
5	(i) Gene expression levels, EC concentrations, age, sex (ii) CNP	(i) CNP (ii) Depression & anxiety	22,195	22,291	Moderate
6	(i) Gene expression levels, EC concentrations, age, sex (ii) Depression & anxiety	(i) Depression & anxiety (ii) CNP	21,810	21,965	Moderate

Abbreviations: AIC, Akaike's information criterion; BIC, Bayesian information criterion.

**FIGURE 3:**
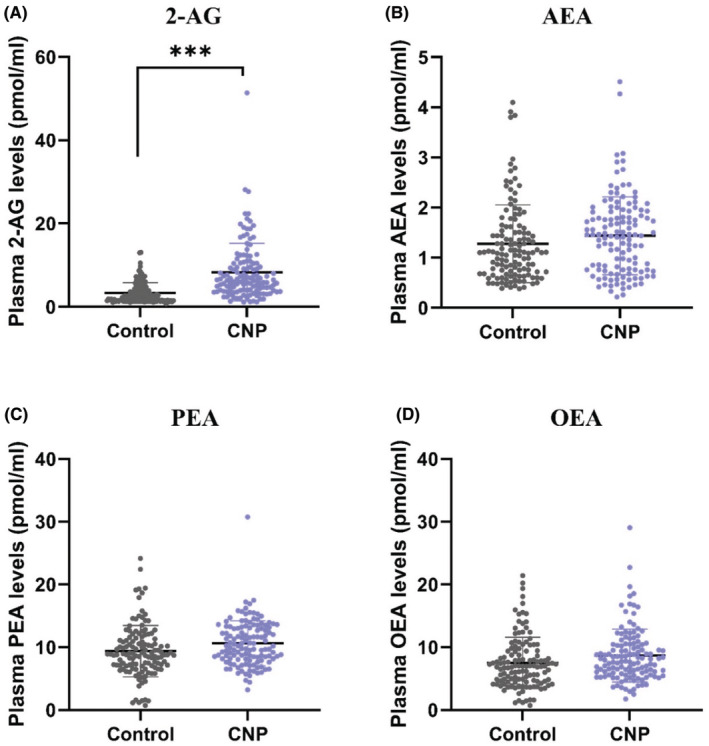
Plasma EC and *N*‐acylethanolamine levels in healthy controls versus patients with CNP (data from both sites combined). (A) 2‐arachidonoylglycerol (2‐AG), (B) anandamide (AEA), (C) *N*‐palmitoylethanolamide (PEA) (D) *N*‐oleoylethanolamide (OEA). *n* = 119–126 per group. ***p < .001 versus control. Data are expressed as mean and standard deviation.

Further, we quantified the levels of the ECs 2‐AG and AEA, and the related *N*‐acylethanolamines, PEA and OEA, in the plasma (Figure 3), and analysed 2‐AG and AEA levels as an outcome of CNP using a multivariate model with age and sex as controlling variables (Analysis 2 in Table 3). The model revealed significant differences (at the 5% significance level) between CNP and control groups to a linear combination of EC levels (Wilk's Λ = .858; *F*_2251_ = 20.7; *p* < .001), controlling for age and sex, with an effect of low‐to‐medium magnitude (partial‐η^2^ = .142). The controlling variable of age, but not sex, was also significantly related at the 5% significance level to a linear combination of outcome measures (Wilk's Λ = .935; *F*_2251_ = 8.71; *p* = .006). Follow‐up univariate analyses revealed that significant differences between CNP and control groups existed on the 2‐AG levels with an effect of low‐to‐medium magnitude (*F*_1252_ = 41.63, *p* < .001, partial‐η^2^ = .142) but not on the AEA levels (*F*_1252_ = 1.27, *p* = .261, partial‐η^2^ = .005) (Figure 3). The levels of 2‐AG were higher in CNP (Marginal mean = 8.2 pmol/mL, 95% Confidence Interval (CI) for Marginal mean = 7.29–9.11) than in the control participants (Marginal mean = 3.78 pmol/mL, 95% CI for Marginal mean = 2.73–4.82).

### 3.4 Higher levels of 2‐AG increased odds of CNP

As the direction of association of CNP with genes and ECs is not clear, we analysed the data using multiple statistical models (Table 3 and Figure S2). The AIC and BIC statistics revealed that the model representing the effect of CNP, age and sex on the ECs (Analysis 2) showed the greatest degree of parsimony of all models tested; that is, this model produced an estimated population matrix that was most consistent with the sample observed covariance matrix (Table 3).

The model of the effect on CNP of expression levels, EC concentrations, age, sex, depression and anxiety was also a relatively well‐fitting model (Analysis 4). The logistic regression model revealed that when controlling for other factors and covariates, expression levels of all EC‐related genes were significantly related (at the 5% significance level) to the outcome of CNP (Table 4). The model revealed that higher 2‐AG concentration (*p* < .001, odds ratio = 1.33, 95% CI, 1.24–1.42), increasing age (*p* < .001, odds ratio = 1.06, 95% CI, 1.04–1.07) and PHQ‐9 scores (*p* < .001, odds ratio = 1·57, 95% CI, 1.47–1.69) increase the odds of CNP. There was no evidence that AEA concentration, sex or STAI‐I score were significantly related (at the 5% significance level) to CNP (Table 4).

**TABLE 4:** Logistic regression analysis of the effect of gene expression, endocannabinoids and clinical characteristics on CNP/control group.

Variable^a^	*p*‐Value	Odds ratio (OR)	95% CI for OR
Gene: *CHPT1*	<.001	.578	(.456, .572)
Gene: *FAAH*	<.001	1.85	(1.46, 2.34)
Gene: *CNR1*	.004	1.35	(1.10, 1.65)
Gene: *DAGLA*	<.001	.403	(.321, .506)
Gene: *MGLL*	<.001	2.21	(1.75, 2.79)
Gene: *NAAA*	<.001	.602	(.463, .781)
Gene: *NAPEPLD*	.012	1.49	(1.09, 2.05)
EC: AEA	.341	1.18	(.837, 1.67)
EC: 2‐AG	<.001	1.33	(1.24, 1.42)
Age	<.001	1.06	(1.04, 1.07)
Sex	.440	1.19	(.763, 1.86)
PHQ‐9	<.001	1.57	(1.47, 1.69)
STAI‐I	.143	1.02	(.993, 1.05)

^a^ For genes, *p*‐values, CI and odds ratio were calculated using ∆Ct values, which are inversely proportionate to gene expression.

## 4 DISCUSSION

Here, we have used an unbiased approach to determine biochemical changes associated with CNP to help understand disease processes. Insights into the CNP disease process may help to identify potential biomarkers as disease indicators or novel analgesic targets that may have clinical utility for better patient care. The S‐LANSS questionnaire is most commonly used for differentiating the likelihood of inflammatory pain versus CNP.^12^ However, comprehensive reviews suggest that the S‐LANSS cannot completely replace thorough patient assessment.^26^, ^27^ In the present study, there was 70.6% and 54.7% concordance of CNP categorisation based on S‐LANSS questionnaires with the clinical diagnosis for cohorts A and B, respectively (Tables S1 and S2). Due to the limitations of S‐LANSS, which have also been described in the literature,^27^ the participants in this study were classified as neuropathic based on their clinical symptomatic diagnoses, irrespective of S‐LANSS scores.

In a previous study, we identified *CHPT1* as one of the top upregulated genes in CNP.^9^ A paucity of studies have investigated CHPT1 in the context of pain. In the current study, we have further confirmed this finding in a combined independent cohort analysis. CHPT1 catalyses PC synthesis by the Kennedy pathway (Figure 1A). PC is a major component of most intracellular membranes and is metabolised into important downstream signalling lipids, such as lyso‐PC, diacylglycerol, and arachidonic acid.^28^ Subsequent metabolites of these lipids, such as prostaglandins, play an important role in the development of chronic pain, notably neuropathic pain.^29^ We expected increased PC concentration in the blood of patients with CNP due to the upregulation of *CHPT1*.^30^ However, we found that PC was significantly lower in CNP plasma compared to the control samples (Figure 1C). Decreased levels of PC have previously been reported in chronic nonspecific low back pain^31^ and in the blood of patients with diabetic neuropathy compared to healthy controls and patients with diabetes without neuropathy.^32^, ^33^ It has been suggested that reduced levels of PC could lead to mitochondrial dysfunction, given the role of PC in energy metabolism and cell structure.^33^, ^34^ A deficit in cellular ATP resulting from impaired functioning of mitochondria is thought to result in a reduction in the activity of the sodium‐potassium pump, resulting in elevated resting membrane potential and spontaneous activity of sensory neurons.^35^ Mitochondrial dysfunction has previously been reported in animal models of nerve injury‐induced neuropathic pain.^36^, ^37^, ^38^

In patients with diabetic neuropathy, lyso‐PC was concurrently increased in serum in addition to decreased levels of PC.^32^ We did not measure lyso‐PC in the present study, but lyso‐PC and its metabolite lysophosphatidic acid have been shown in preclinical models to contribute to the development of neuropathic pain.^39^, ^40^ Lyso‐PC synthesis is upregulated following nerve injury resulting from the release of the excitatory neurotransmitters substance P and glutamate from primary afferent neurons or the increase in reactive oxygen species.^41^ Furthermore, a significant association between lysophosphatidic acid and both pain intensity and symptoms has previously been demonstrated in a heterogeneous group of patients with neuropathic pain.^42^ Interestingly, in the sciatic nerve transection model of neuropathic pain, PC was increased, and lyso‐PC was decreased 5 days after the induction of the model.^43^, ^44^ Therefore, it is possible that the lower blood levels of PC in patients with CNP in the present study could be due to its fast utilisation and conversion into downstream signalling molecules, thus causing a sustained overexpression of *CHPT1* as a compensatory mechanism. The upregulation of *CHPT1* could ultimately lead to a number of molecular cascades, which could promote the development of neuropathic pain. This hypothesis led us to explore downstream associated pathways of PC metabolism.

One of the CNP‐related pathways of PC catabolism is the synthesis of ECs (Figure 2A). ECs are synthesised ‘on demand’ by hydrolysis of cell membrane phospholipid precursors.^45^ They mediate their effect by binding and activating cannabinoid CB_1_ (*CNR1*) and CB_2_ (*CNR2*) receptors.^46^ The mRNA expression analysis showed significant downregulation of *MGLL* and *NAPEPLD* in CNP (Figure 2B). No significant effect of CNP on the expression of *CNR1* was observed. The levels of *CNR2* mRNA were very low in the tested samples and could not be analysed. Further analysis using LC–MS/MS revealed significantly higher plasma levels of 2‐AG in patients with CNP compared to the control group (Figure 3). This corroborates the gene expression analysis because *MGLL*, which was downregulated, encodes for the principal hydrolysing enzyme of 2‐AG. The levels of AEA, PEA and OEA also showed a strong trend in the same direction despite less robust alterations in the expression of genes encoding their metabolising enzymes (Figures 2 and 3). The endocannabinoid system is well‐positioned to modulate pain. 2‐AG and AEA bind to CB_1_ and CB_2_ receptors expressed at peripheral, spinal, and supraspinal sites to mediate antinociception.^47^, ^48^ Cannabinoid‐mediated antinociception likely results from several mechanisms, including but not limited to the inhibition of presynaptic neurotransmitter and neuropeptide release, modulation of postsynaptic neuronal excitability, and activation of the descending inhibitory pain pathway.^47^, ^49^ In preclinical studies, inhibition of FAAH and MGL, the catabolic enzymes for AEA and 2‐AG, respectively, significantly attenuated mechanical and cold hypersensitivity in models of neuropathic pain.^23^, ^48^, ^49^, ^50^, ^51^, ^52^

Clinical evidence supporting the role of the endocannabinoid system in pain includes an interesting case study where a microdeletion in a dorsal root ganglia and brain‐expressed pseudogene, FAAH‐OUT, together with a single nucleotide polymorphism in the gene responsible for the transcription of FAAH, resulted in reduced catabolic activity and therefore, an increase in circulating AEA levels. The individual carrying these mutations described markedly lower levels of pain and anxiety and no requirement for postoperative analgesia after a normally painful orthopaedic hand surgery.^53^ In patients with painful diabetic neuropathy, it was found that serum AEA levels were higher compared to patients with painless diabetic neuropathy.^54^ In that study, levels of ECs were not compared to healthy controls; therefore, direct comparison with the present study is not possible. However, elevated plasma levels of both AEA and 2‐AG have been reported in patients with neuromyelitis optica.^55^ Furthermore, in that study, 2‐AG was negatively correlated with mechanical pain thresholds, whereas higher 2‐AG levels were associated with lower pain sensitivity. Thus, based on these data, an increase in ECs likely represents a compensatory mechanism in CNP, as also suggested by the statistical analysis (Analysis 2 in Table 3).

The statistical model formed using a combination of age, sex, depression scores, anxiety scores, expression of genes related to EC metabolism and EC levels revealed that increase in age, 2‐AG levels, PHQ‐9 and STAI‐I scores were associated with higher odds of CNP (Table 4). Depression and anxiety are common comorbidities associated with CNP due to the overlap between molecular and neuroplasticity changes in these conditions.^56^, ^57^, ^58^, ^59^ In the present study, the PHQ‐9 and STAI‐I scores were higher in patients with CNP than control participants (Table 2). In fact, 75% of the patient cohort were classified as having mild to severe depression versus 12.8% of healthy controls. *CHPT1* has also been found to be upregulated in patients with ovarian cancer with concurring depression but not in patients without depression.^60^ A previous report has also found that in patients with major depression, serum levels of 2‐AG are significantly decreased compared to controls.^61^ It is possible that the interaction between depression and pain in the present cohort contributed to the manifestation of multiple alterations within related pathways, possibly in an attempt to restore homeostasis. Indeed, preclinical studies using inhibitors of hydrolysing enzymes such as FAAH and MGL have shown that ECs have beneficial effects on CNP and depression.^23^, ^62^ However, the role of *CHPT1* in pain and depression is yet to be elucidated.

Alterations in plasma lipids have been observed during or following stress and are also associated with anxiety and depression.^63^, ^64^ Indeed, one study revealed an inverse correlation between plasma PC levels and depression and anxiety symptoms, assessed using the Hospital Anxiety and Depression scale.^64^ However, modification of the plasma lipids associated with the Kennedy pathway and concurring alterations in the endocannabinoid system could be specific to, and have the potential as a biomarker of, CNP.

Understanding the relationship between circulating ECs and central processes remains a major gap in the literature. Currently, there is little evidence to support the idea that changes in peripheral levels are a result of central nervous system spillover. While the source of circulating ECs is unclear, immune cells and the response of non‐immune cells to nerve injury could critically contribute to their levels in blood.^65^, ^66^ 2‐AG is synthesised and released on demand following stimuli of different origins, including stress and pain. 2‐AG is involved in the bidirectional modulation of stress‐depression‐chronic pain and immune system regulation. In response to injury, immune cells in the periphery produce 2‐AG, which functions to reduce the inflammatory response of these cells, primarily via activation of CB_2_ receptors.^67^ Thus, the increase in plasma levels of ECs may reflect a compensatory increase aimed at regulating pain levels.

A limitation of this study is the inherent heterogeneity of the human CNP samples due to medications and any underlying comorbidity. We could not include variables related to medications in the statistical analysis as the cohorts were not balanced for these metrics and the sample size was not sufficiently large for any further analysis.

## 5 CONCLUSION

The screening of CNP is currently largely based on clinical evaluations and questionnaires such as the S‐LANSS. However, there is a fundamental need to identify novel biomarkers for CNP that could function as both novel analgesic drug targets and specific disease indicators for improved patient care. In the present study, we demonstrated alterations in both molecular and biochemical markers in patients with CNP compared to healthy controls in an independent combined cohort analysis. The elucidation of these underlying processes associated with CNP could represent important biomarkers in clinical utility for patient care.

## AUTHOR CONTRIBUTIONS

Conceptualisation: DPF and PCMH; Data Curation: SLB, BI, JS, DPF and PCMH; Funding acquisition: DPF and PCMH; Investigation: SLB, BI, ESG, JS, DPF and PCMH, Supervision: DPF and PCMH; writing‐original draft: SLB, BI, JS, DPF and PCMH; writing–review and editing: SLB, ESG, BI, JS, DPF and PCMH.

## FUNDING INFORMATION

This work was supported by grants from the Pain Relief Foundation, British Pain Society Clulow Award and the Irish Research Council Postgraduate Scholarship (GOIPG/2019/3945).

## CONFLICT OF INTEREST STATEMENT

All authors declare no conflicts of interest.

## Supporting information


Appendix S1.

Appendix S2.

## Data Availability

The datasets supporting the conclusions of this article are included within the article and supplementary material.
