# Supplementary material for: Clinical measures in chronic neuropathic pain are related to the Kennedy and endocannabinoid pathways
Source: Eur J Clin Invest. 2024 Nov 15;55(2):e14351. doi: 10.1111/eci.14351 (PMC11744925; doi:10.1111/eci.14351)
Supplement: Supplementary file 1 — Appendix S1. [file ECI-55-e14351-s002.docx]

**SUPPLEMENTARY METHODS**

**Blood processing for Quantitative Real Time-PCR (qRT-PCR) analysis**

| **Gene** | **Forward 5' -> 3'** | **Reverse 5' -> 3'** |
| --- | --- | --- |
|  |  |  |
| *CHPT1* | AGCTCTTTGACCATGGCTGT | TAAGTTCCTAAGCGAGCGGC |
| *DAGLA* | CTACACCTCCTGCAACGAC | TTACGCTGCCTCCTCTTG |
| *FAAH* | GAGAAGAGGTCTACACCAGC | GGGGTATGTTGCTTGGC |
| *NAPEPLD* | AAGATCACAGCAGTGTTCCA | ACCATTACCGTGGCATGTC |
| *NAAA* | ACGACTTGGACTTGGTGC | GGTTGACCAGAAGGCAGTC |
| *CNR1* | GGTAAGACCTGGCAGAGTT | CGGAAGGTGGTATCTGCAA |
| *MGLL* | CCCTCATCTTTGTGTCCCA | GTGGAAGTCAGACACTACCAT |
| *GAPDH* | PRIMER DESIGN REFERENCE GENE/GENORM 600rx | |
| *SDHA* | PRIMER DESIGN REFERENCE GENE/GENORM 600rx | |
| *ACTB* | PRIMER DESIGN REFERENCE GENE/GENORM 600rx | |

Blood from all participants were collected using PAXgene® tubes (Qiagen, Manchester, UK), the tubes were inverted 10x and stored -80 ^o^C until use. Total RNA was extracted using the PAXgene® RNA extraction kit according to the manufacturer’s instructions (Qiagen). In brie f, the RNA was treated with DNase (Thermo Scientific™, Loughborough, UK) and purified on columns. The final RNA concentration was measured on a NanoDrop ND2000 ultraviolet–visible spectrophotometer (Labtech International Ltd, UK). For qRT-PCR A total of 300 ng of RNA from each human sample was reverse transcribed using the Verso cDNA Synthesis Kit (Thermo Scientific™) according to the manufacturer’s instructions. The cDNA was subsequently diluted 10-fold. Amplification was performed in triplicate with a Roche LightCycler® 480 II (Roche Diagnostics Ltd., Burgess Hill, West Sussex, United Kingdom). Each 10 μl reaction mixture contained 3 μl of LightCycler^®^ 480 SYBR Green I Master (Roche Diagnostics, Mannheim, Germany), 300 nM of each forward and reverse primer (described below) and 1 μl of diluted cDNA. Amplification protocol was as follows: Polymerase activation and DNA denaturation at 95 °C for 2 min, 40 cycles of denaturation at 95 °C for 5 s with annealing and extension at 60 °C for 30 s followed by fluorescence detection. Upon completion of thermal cycling, a melt-curve analysis was carried out to confirm reaction specificity. Before the analysis of selected genes, a geNorm analysis was performed on 12 samples to determine the most stably expressed genes. *GAPDH, ACTB* and *SDHA* were determined as the reference genes (M values (gene expression stability measure) <0·5, CV (Coefficient of Variation) <25%). The relative gene expression of the markers was normalised to the geometric mean of *GAPDH, ACTB* and *SDHA* and then compared to the control group, according to the 2^–ΔΔCt^ method. The following primers were used for the qRT-PCR of the tested genes:

**Plasma Collection**

BD Vacutainer K2-EDTA (BD Biosciences tubes, Berkshire, UK) were used for the collection of plasma from the participants. The tubes were inverted 10x, centrifuged at 1000*×g* for 10 min, and plasma was Snap frozen on dry ice and stored immediately at −80 °C. Samples were thawed on ice just before use for estimation of phosphatidylcholine and endocannabinoids.

**Endocannabinoid Quantification by HPLC – Tandem Mass Spectrometry**

BD Plasma samples were centrifuged at 1,400g for 15 minutes at 4°C prior to transferring 200 µl of plasma into a 1.5 ml microfuge tube. Plasma samples were then spiked with 20 µl of internal standard mix (containing 50 ng of 2-AG-d8, 2·5 ng of AEA-d8, 2·5 ng of OEA-d4 and 2·5 ng of PEA-d4). Samples were then vortex-mixed and allowed to equilibrate for 10 minutes on ice. For protein precipitation, 1 ml of acetonitrile (ACN) at 4°C containing 0·1% formic acid was added to each sample followed by incubation on ice for a further 30 minutes. The precipitated proteins were pelleted by centrifugation at 1,400g for 15 minutes at 4°C. A filter (Fisherbrand^TM^ Non-sterile PTFE Hydrophyl, 25 mm, 0·45 µM Syringe Filter, Fisher Scientific, Ireland) was placed in a 5 ml SafeSeal tube (SARSTEDT, Ireland). The plunger/piston from a 1 ml syringe was removed and the syringe was attached to the filter by carefully inserting the syringe neck into the filter inlet. The piston is retained and stored by placing in a clean microfuge tube. 1100 µl of the supernatant was removed, with care taken so as not to disturb the pellet, and loaded into the syringe. The supernatant was allowed to flow through the filter until the syringe emptied. 700 µl of ACN were added to the syringe to displace the dead volume of the filter. The plunger of the syringe was slowly re-inserted and the ACN was then pushed through the filter. The collected eluate was vortex-mixed and then 500 µl were transferred to a new 1·5 ml microfuge tube and dried down at 45°C for ~1 hr in a centrfigual concentrator (Eppendorf Concentrator plus complete system, Davidson and Hardy Ltd, Ireland) and re-suspended in 40 µl of 100 % ACN before transferring to HPLC vials.

Mobile phases consisted of (A) HPLC grade water with 0·1% (v/v) formic acid and (B) ACN with 0·1% (v/v) formic acid with a flow rate of 0.2 ml/min using a Phenomenex Synergi™ Fusion-RP 80 Å C18 column - (4 µm particles, 50 mm length, 2 mm diameter). A reverse phase gradient elution was used, comprising 45% solution B for the first minute, then linearly increased to 100% solution B until 5 minutes into the run and maintained at 100% solution B until the assay run finished at 12 minutes. A further 4·7 minutes was required to re-equilibrate the column at 45% solution B before the next injection. Under these conditions, AEA, 2-AG, PEA, OEA eluted at the following retention times: 5·7, 6·0, 6·3 and 6·5 minutes, respectively. Analyte detection was carried out in electrospray-positive ionisation mode on an Agilent 1260 infinity 2 HPLC system coupled to a SCIEX QTRAP 4500 mass spectrometer operated in triple quadrupole mode (SCIEX Ltd, Phoenix House Lakeside Drive Centre Park, United Kingdom). Instrument conditions were optimised for each analyte by infusing standards separately. Quantitation of target endocannabinoids was achieved by positive ion electrospray ionization and multiple reaction monitoring (MRM) mode, allowing simultaneous detection of the protonated precursor and product molecular ions [M+ H+] of the analytes of interest and the deuterated form of the internal standard. Quantitation of each analyte was performed by determining the peak area response of each target analyte against its corresponding deuterated internal standard. This ratiometric analysis was calculated using Skyline Quantitative Analysis Software, version 4.2 (MacCoss Lab Software, University of Washington, USA). The amount of analyte in unknown samples was calculated from the analyte/internal standard peak area response ratio using a 10-point calibration curve constructed from a range of concentrations of the non-deuterated form of each analyte and a fixed amount of deuterated internal standard. The values obtained from the Skyline Analysis Software are initially expressed in ng per 200 µL of plasma. To express values as nmol/µL the corresponding values were divided by 200, then divided by the molar mass of each analyte, and then finally the nmol/µL values were multiplied by 1000 to express as pmol/mL.
