# Supplementary material for: Clinical measures in chronic neuropathic pain are related to the Kennedy and endocannabinoid pathways
Source: Eur J Clin Invest. 2024 Nov 15;55(2):e14351. doi: 10.1111/eci.14351 (PMC11744925; doi:10.1111/eci.14351)
Supplement: Supplementary file 2 — Appendix S2. [file ECI-55-e14351-s001.docx]

**SUPPLEMENTARY FIGURES**


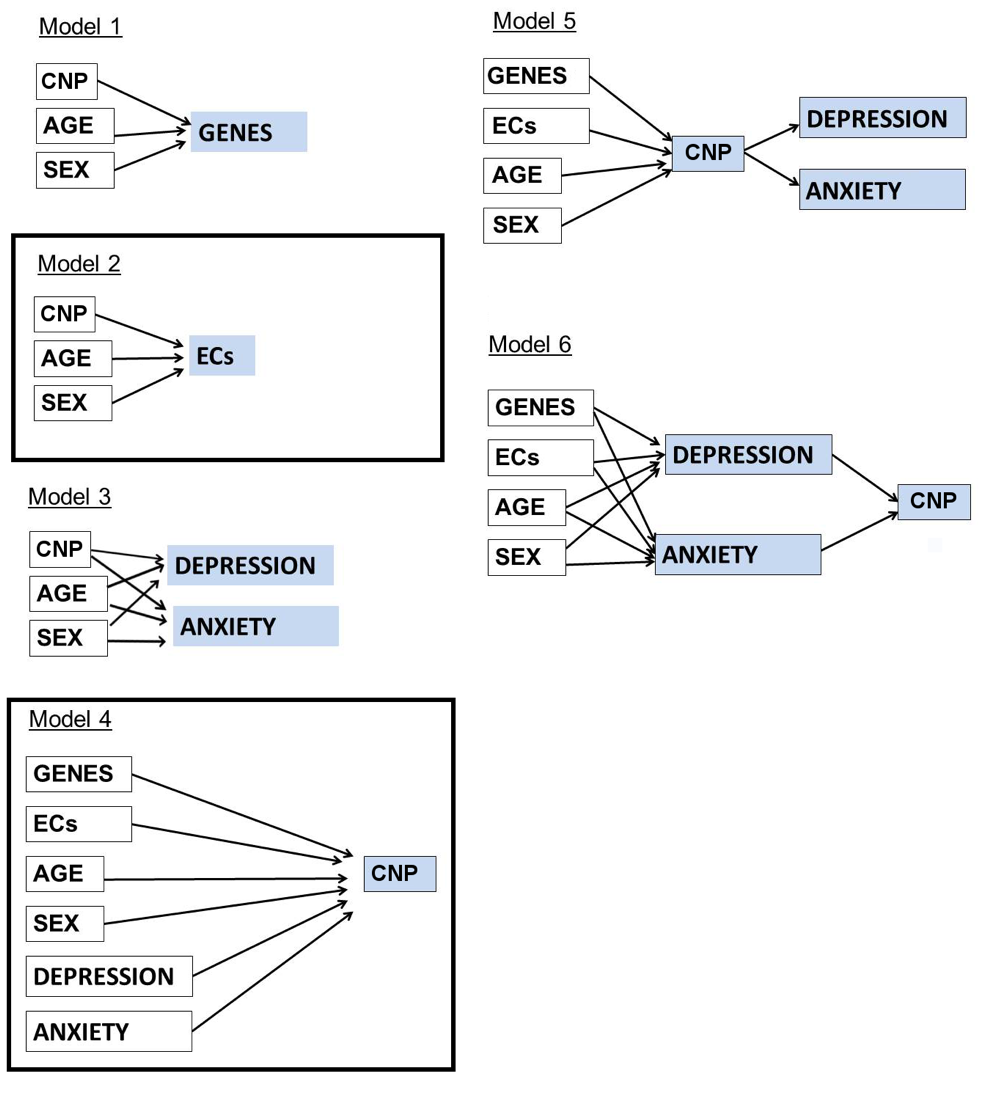


**Fig. S1**. Statistical models for the association of CNP with gene expression (genes), endocannabinoids (ECs), age, sex, depression and anxiety. Depression and anxiety were measured using PHQ-9 and STAI-I scores, respectively. The models 1 to 6 are derived from analysis 1 to 6, respectively. The models in the boxes show a good fit and are discussed in the main text.


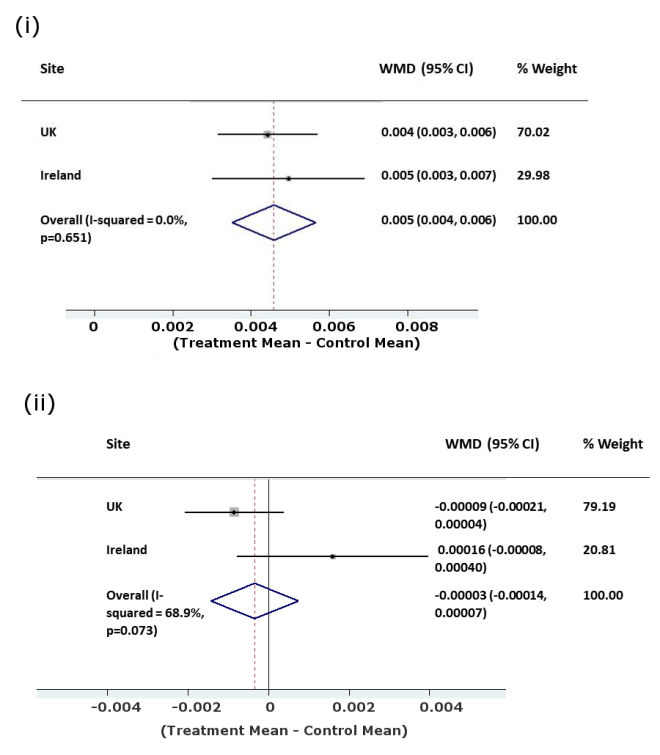


**Fig. S2**. Forest plots to evaluate fixed-effect meta-analyses of site-level primary outcomes for (i) 2-AG and (ii) anandamide (AEA). The plots showed similar group effects across sites for 2-AG, but moderate heterogeneity across sites for AEA. Overall, the evidence for heterogeneity across sites was inconclusive.

**Table S1.** Clinical characteristics*^a^* of samples in cohort A; CNP and control samples codes start with NPL and CHL, respectively.

**SUPPLEMENTARY TABLES**

| **CODE** | **AGE** | **GENDER**  **CODE*^b^*** | **DURATION**  **(months)** | **SLANSS** | **PHQ9** | **CPG** | **STAI-I** | **2-AG (pmol/μL)** | **AEA (pmol/μL)** | **PEA (pmol/μL)** | **OEA (pmol/μL)** | **GENE*^c^*** | **PC (mM)** | **ANTIINF*^d^*** | **ANTIDEP**^d^ | **ANTICONV*^d^*** | **OPIOIDS** |  |
| --- | --- | --- | --- | --- | --- | --- | --- | --- | --- | --- | --- | --- | --- | --- | --- | --- | --- | --- |
| NPL001 | 68 | 1 | 24 | 0 | 4 | II | 44 | 0·0068037 | 0·0007775 | 0·0080789 | 0·0076624 | Yes | 3·22441558 | 0 | 0 | 1 | 0 |  |
| NPL002 | 64 | 1 | 36 | 17 | 6 | II | 23 | 0·0035994 | 0·0004499 | 0·0051561 | 0·0055637 | Yes | 0·93558442 | 1 | 1 | 0 | 0 |  |
| NPL003 | 40 | 2 | 84 | 16 | 13 | II | 45 | 0·0073351 | 0·0003446 | 0·0053398 | 0·0028653 | Yes | 3·40883117 | 0 | 1 | 1 | 0 |  |
| NPL004 | 38 | 2 | 36 | 23 | 10 | IV | 36 | 0·0066665 | 0·0006214 | 0·0047361 | 0·0050722 | Yes | 0·7974026 | 0 | 1 | 1 | 0 |  |
| NPL005 | 48 | 2 | 108 | 18 | 11 | IV | 42 | 0·0028092 | 0·0004085 | 0·0064325 | 0·0055565 | Yes | NR | 0 | 1 | 0 | 0 |  |
| NPL006 | 49 | 1 | 60 | 17 | 14 | II | 39 | 0·007312 | 0·0003027 | 0·004524 | 0·0033034 | Yes | 2·380779221 | 1 | 1 | 1 | 0 |  |
| NPL007 | 21 | 1 | 18 | 13 | 12 | III | 56 | 0·0110054 | 0·0007022 | 0·0037411 | 0·0055539 | Yes | 0·312727273 | 0 | 0 | 0 | 1 |  |
| NPL008 | 68 | 2 | 120 | 19 | 7 | III | 39 | 0·0052336 | 0·0002391 | 0·0046526 | 0·0021296 | Yes | NR | 0 | 0 | 1 | 1 |  |
| NPL009 | 56 | 1 | 96 | 5 | 3 | I | 26 | 0·0088966 | 0·0006941 | 0·0057574 | 0·0043095 | Yes | NR | 1 | 0 | 1 | 0 |  |
| NPL010 | 40 | 2 | 38 | 22 | 25 | IV | 67 | 0·0049313 | 0·0005006 | 0·0053353 | 0·0053153 | Yes | 1·04623377 | 0 | 0 | 0 | 0 |  |
| NPL011 | 60 | 1 | 228 | 10 | 6 | III | 49 | 0·021193 | 0·0005578 | 0·0069334 | 0·0044887 | Yes | 3·76103896 | 1 | 0 | 0 | 0 |  |
| NPL012 | 44 | 2 | 24 | 17 | 16 | IV | 43 | 0·0041379 | 0·0005741 | 0·0046395 | 0·0043966 | Yes | 0·78077922 | 1 | 0 | 1 | 0 |  |
| NPL013 | 42 | 2 | 24 | 6 | 12 | IV | 60 | 0·0123009 | 0·0002005 | 0·0025335 | 0·0015128 | Yes | 0·32363636 | 0 | 0 | 0 | 0 |  |
| NPL014 | 49 | 2 | 120 | 4 | 8 | IV | 63 | 0·0033994 | 0·0005187 | 0·0048984 | 0·0034031 | Yes | 1·55948052 | 0 | 0 | 0 | 0 |  |
| NPL015 | 59 | 2 | 132 | 19 | 18 | IV | 32 | 0·0022332 | 0·0006156 | 0·0060137 | 0·0056207 | Yes | 1·44207792 | 0 | 0 | 0 | 0 |  |
| NPL016 | 40 | 1 | 168 | 24 | 19 | III | 71 | 0·0076138 | 0·0005747 | 0·004959 | 0·0043993 | Yes | 2·04779221 | 0 | 1 | 0 | 0 |  |
| NPL017 | 74 | 1 | 588 | 0 | 6 | IV | 26 | 0·0039378 | 0·0007012 | 0·0051077 | 0·0044216 | Yes | 0·14649351 | 1 | 1 | 0 | 0 |  |
| NPL018 | 40 | 2 | 192 | 23 | 16 | IV | 53 | 0·0058266 | 0·0011368 | 0·0076648 | 0·0065838 | Yes | NR | 0 | 1 | 0 | 1 |  |
| NPL019 | 36 | 2 | 84 | 24 | 18 | IV | 58 | 0·0030547 | 0·0005253 | 0·0068973 | 0·004807 | Yes | NR | 1 | 0 | 0 | 0 |  |
| NPL020 | 37 | 1 | 42 | 18 | 14 | IV | 45 | 0·0091248 | 0·0008205 | 0·0056189 | 0·004811 | Yes | NR | 0 | 0 | 0 | 0 |  |
| NPL021 | 41 | 1 | 24 | 12 | 19 | III | 64 | 0·0039456 | 0·0009031 | 0·0075588 | 0·0059038 | Yes | 1·30493506 | 0 | 1 | 1 | 0 |  |
| NPL022 | 48 | 1 | 18 | 24 | 21 | IV | 55 | 0·0062321 | 0·000627 | 0·0057114 | 0·0052727 | Yes | NR | 1 | 0 | 0 | 0 |  |
| NPL023 | 43 | 2 | 36 | 9 | 20 | IV | 69 | 0·0073883 | 0·0013827 | 0·0121346 | 0·0091286 | Yes | NR | 0 | 0 | 0 | 1 |  |
| NPL024 | 50 | 2 | 13 | 5 | 1 | I | 21 | 0·001163 | 0·0008729 | 0·0072738 | 0·0061894 | Yes | NR | 1 | 0 | 0 | 1 |  |
| NPL025 | 37 | 2 | 36 | 2 | 20 | IV | 56 | 0·004425 | 0·0008966 | 0·0073173 | 0·0054339 | Yes | NR | 1 | 0 | 0 | 1 |  |
| NPL026 | 61 | 1 | 240 | 24 | 23 | IV | 27 | 0·0036244 | 0·00042 | 0·0063738 | 0·0041401 | Yes | NR | 1 | 1 | 1 | 1 |  |
| NPL027 | 51 | 1 | 48 | 24 | 25 | IV | 40 | 0·0088969 | 0·0006722 | 0·0064859 | 0·0049623 | Yes | NR | 0 | 0 | 1 | 1 |  |
| NPL028 | 39 | 1 | 96 | 15 | 21 | III | 48 | 0·0094629 | 0·0007682 | 0·0068762 | 0·0046836 | Yes | NR | 0 | 0 | 0 | 1 |  |
| NPL029 | 43 | 2 | 84 | 18 | 3 | II | 27 | 0·0035535 | 0·0005267 | 0·0054467 | 0·0038585 | Yes | NR | 1 | 1 | 0 | 1 |  |
| NPL030 | 49 | 1 | 72 | 24 | 2 | II | 23 | 0·0066629 | 0·0004431 | 0·0043037 | 0·0028307 | Yes | NR | 0 | 1 | 0 | 1 |  |
| NPL031 | 48 | 2 | 42 | 19 | 12 | IV | 45 | 0·0064794 | 0·0006837 | 0·005608 | 0·0040228 | Yes | 1·53454545 | 1 | 1 | 1 | 1 |  |
| NPL032 | 68 | 2 | 24 | 22 | 8 | IV | 31 | 0·0054906 | 0·0008053 | 0·0064469 | 0·0045017 | Yes | 1·69454545 | 0 | 1 | 0 | 1 |  |
| NPL033 | 34 | 1 | 204 | 19 | 20 | IV | 61 | 0·0053444 | 0·0010118 | 0·0062468 | 0·0075832 | Yes | 0·64675325 | 0 | 1 | 1 | 1 |  |
| NPL034 | 24 | 2 | 36 | 19 | 17 | IV | 51 | 0·004654 | 0·0004355 | 0·0035028 | 0·0030836 | Yes | 0·94597403 | 1 | 0 | 1 | 1 |  |
| NPL035 | 27 | 2 | 54 | 20 | 16 | IV | 56 | 0·0028243 | 0·0007186 | 0·0060362 | 0·0059216 | NR | NR | NR | NR | NR | NR |  |
| NPL037 | 45 | 2 | 180 | 19 | 21 | III | 65 | 0·004732 | 0·0012758 | 0·0074847 | 0·0081302 | NR | 1·20467532 | 0 | 0 | 0 | 1 |  |
| NPL038 | 51 | 2 | 156 | 18 | 14 | IV | 66 | 0·0121965 | 0·0015809 | 0·0095042 | 0·0107137 | Yes | NR | 1 | 0 | 1 | 1 |  |
| NPL039 | 48 | 2 | 132 | 16 | 14 | I | 47 | 0·0018542 | 0·0003735 | 0·0048966 | 0·0025273 | Yes | NR | 0 | 0 | 1 | 1 |  |
| NPL040 | 45 | 1 | 18 | 3 |  | NR | 57 | 0·01244 | 0·0003816 | 0·006247 | 0·0044151 | NR | NR | 0 | 0 | 1 | 1 |  |
| NPL041 | 33 | 1 | 96 | 24 | 11 | III | 48 | 0·0075592 | 0·0005448 | 0·0077526 | 0·0052109 | Yes | NR | 1 | 1 | 1 | 1 |  |
| NPL042 | 43 | 1 | 72 | 17 | 17 | IV | 38 | 0·0104089 | 0·0006997 | 0·0067114 | 0·0045775 | Yes | NR | 1 | 0 | 1 | 1 |  |
| NPL043 | 38 | 2 | 180 | 24 | 9 | III | 54 | 0·0039193 | 0·0012963 | 0·0087631 | 0·0081939 | Yes | NR | 1 | 0 | 0 | 1 |  |
| NPL044 | 37 | 2 | 54 | 2 | 21 | III | 50 | 0·0167255 | 0·0006654 | 0·0091781 | 0·0064384 | Yes | NR | 0 | 1 | 1 | 1 |  |
| NPL045 | 47 | 2 | 108 | 14 | 11 | IV | 40 | 0·0032278 | 0·000546 | 0·0084488 | 0·0052005 | Yes | NR | 1 | 1 | 1 | 1 |  |
| NPL046 | 54 | 1 | 24 | 0 | 0 | II | 37 | 0·0163854 | 0·0007539 | 0·0091333 | 0·0050722 | Yes | NR | 0 | 1 | 1 | 1 |  |
| NPL047 | 49 | 1 | 18 | 3 | 3 | I | 32 | 0·00556 | 0·001125 | 0·0070882 | 0·0065627 | Yes | NR | 1 | 0 | 0 | 1 |  |
| NPL048 | 21 | 2 | 11 | 24 | 21 | IV | 37 | 0·0029804 | 0·0005381 | 0·0051657 | 0·0033408 | Yes | NR | 1 | 1 | 0 | 1 |  |
| NPL049 | 79 | 1 | 48 | 7 | 3 | II | 46 | 0·017063 | 0·0013715 | 0·0102613 | 0·0096048 | Yes | NR | 1 | 0 | 0 | 1 |  |
| NPL050 | 50 | 2 | 300 | 24 | 24 | IV | 59 | NR | NR | NR | NR | Yes | NR | 1 | 0 | 1 | 1 |  |
| NPL052 | 29 | 2 | 30 | 24 | 26 | IV | 51 | 0·00188 | 0·0004179 | 0·0051579 | 0·0034452 | Yes | NR | 1 | 1 | 1 | 1 |  |
| NPL053 | 44 | 1 | 204 | 15 | 4 | IV | 33 | 0·0091124 | 0·000922 | 0·0076418 | 0·0066998 | Yes | NR | 0 | 0 | 1 | 1 |  |
| CHL01 | 45 | 2 |  |  | 2 |  | 21 | 0·0029102 | 0·0007776 | 0·0059197 | 0·0055655 | Yes | 5·42597403 |  |  |  |  |  |
| CHL02 | 53 | 2 |  |  | 1 |  | 23 | 0·0015327 | 0·0003788 | 0·0047809 | 0·0033444 | Yes | NR |  |  |  |  |  |
| CHL03 | 22 | 2 |  |  | 0 |  | 22 | 0·0019837 | 0·0010207 | 0·0064898 | 0·0064076 | Yes | 4·9412987 |  |  |  |  |  |
| CHL04 | 61 | 2 |  |  | 2 |  | 23 | 0·0012749 | 0·0004374 | 0·0038419 | 0·0031442 | Yes | NR |  |  |  |  |  |
| CHL05 | 27 | 2 |  |  | 4 |  | 48 | 0·001537 | 0·0010228 | 0·0075689 | 0·0075237 | Yes | 7·54337662 |  |  |  |  |  |
| CHL06 | 52 | 1 |  |  | 0 |  | 20 | 0·0086953 | 0·0009621 | 0·0085938 | 0·0054835 | Yes | NR |  |  |  |  |  |
| CHL07 | 43 | 1 |  |  | 2 |  | 31 | 0·0040398 | 0·0016329 | 0·0099277 | 0·0093236 | Yes | NR |  |  |  |  |  |
| CHL08 | 49 | 2 |  |  | 0 |  | 25 | 0·0032054 | 0·0016505 | 0·0101292 | 0·0118554 | Yes | NR |  |  |  |  |  |
| CHL09 | 21 | 2 |  |  | 2 |  | 41 | 0·0016936 | 0·0006954 | 0·00691 | 0·0057934 | Yes | NR |  |  |  |  |  |
| CHL10 | 24 | 1 |  |  | 8 |  | 24 | 0·0085145 | 0·0010088 | 0·006943 | 0·0048295 | Yes | NR |  |  |  |  |  |
| CHL11 | 62 | 2 |  |  | 0 |  | 20 | 0·0019523 | 0·0007971 | 0·0081538 | 0·0063496 | Yes | NR |  |  |  |  |  |
| CHL12 | 23 | 2 |  |  | 2 |  | 33 | 0·0028433 | 0·0006915 | 0·0070796 | 0·0064312 | Yes | NR |  |  |  |  |  |
| CHL13 | 25 | 1 |  |  | 7 |  | 35 | 0·0027014 | 0·0013796 | 0·0080753 | 0·0063767 | Yes | NR |  |  |  |  |  |
| CHL14 | 46 | 1 |  |  | 8 |  | 34 | 0·0026515 | 0·000579 | 0·0057382 | 0·004193 | Yes | NR |  |  |  |  |  |
| CHL15 | 21 | 2 |  |  | 0 |  | 20 | 0·0026581 | 0·0005742 | 0·0089532 | 0·0039524 | Yes | 4·14441558 |  |  |  |  |  |
| CHL16 | 30 | 2 |  |  | 1 |  | 25 | 0·0029423 | 0·0004577 | 0·0051329 | 0·0038488 | Yes | 5·19636364 |  |  |  |  |  |
| CHL17 | 21 | 2 |  |  | 8 |  | 50 | 0·0022771 | 0·0005196 | 0·005563 | 0·0036565 | Yes | 5·29142857 |  |  |  |  |  |
| CHL18 | 26 | 2 |  |  | 3 |  | 34 | 0·0031587 | 0·0003554 | 0·0046794 | 0·0025306 | Yes | NR |  |  |  |  |  |
| CHL19 | 51 | 2 |  |  | 0 |  | 24 | 0·0033077 | 0·000347 | 0·0052005 | 0·0030132 | Yes | NR |  |  |  |  |  |
| CHL20 | 24 | 1 |  |  | 0 |  | 20 | 0·0054118 | 0·0007956 | 0·0060985 | 0·0053115 | Yes | NR |  |  |  |  |  |
| CHL21 | 48 | 1 |  |  | 0 |  | 20 | 0·0047448 | 0·0008022 | 0·0069064 | 0·0051586 | Yes | NR |  |  |  |  |  |
| CHL22 | 21 | 1 |  |  | 5 |  | 42 | 0·0016504 | 0·0009644 | 0·0068794 | 0·0051507 | Yes | NR |  |  |  |  |  |
| CHL23 | 18 | 1 |  |  | 3 |  | 33 | 0·0010499 | 0·0017201 | 0·011708 | 0·0132085 | Yes | 2·66337662 |  |  |  |  |  |
| CHL24 | 51 | 2 |  |  | 1 |  | 32 | 0·0077084 | 0·0005501 | 0·0051323 | 0·0035074 | Yes | 8·55896104 |  |  |  |  |  |
| CHL25 | 23 | 2 |  |  | 1 |  | 20 | 0·0014115 | 0·0018259 | 0·0106894 | 0·0137332 | Yes | 6·9787013 |  |  |  |  |  |
| CHL26 | 25 | 2 |  |  | 2 |  | 20 | 0·0015691 | 0·0006031 | 0·005636 | 0·0037969 | Yes | 2·99220779 |  |  |  |  |  |
| CHL27 | 21 | 1 |  |  | 1 |  | 22 | 0·001305 | 0·0011428 | 0·0056723 | 0·006252 | Yes | NR |  |  |  |  |  |
| CHL28 | 21 | 2 |  |  | 4 |  | 36 | 0·001488 | 0·0011726 | 0·0075344 | 0·0072088 | Yes | NR |  |  |  |  |  |
| CHL29 | 51 | 1 |  |  | 5 |  | 31 | 0·0028595 | 0·0005982 | 0·0066207 | 0·0047664 | Yes | NR |  |  |  |  |  |
| CHL30 | 41 | 2 |  |  | 5 |  | 32 | 0·0020502 | 0·0005372 | 0·0058939 | 0·0028307 | NR | 10·134026 |  |  |  |  |  |
| CHL31 | 53 | 2 |  |  | 2 |  | 29 | 0·0012267 | 0·0004843 | 0·0055803 | 0·0040228 | NR | 5·27428571 |  |  |  |  |  |
| CHL32 | 45 | 2 |  |  | 2 |  | 25 | 0·0056702 | 0·0007191 | 0·0049904 | 0·0042818 | Yes | 7·61298701 |  |  |  |  |  |
| CHL34 | 48 | 2 |  |  | 0 |  | 22 | 0·0016316 | 0·0011759 | 0·0082465 | 0·0068595 | Yes | 7·30493506 |  |  |  |  |  |
| CHL35 | 68 | 2 |  |  | 0 |  | 21 | 0·0013113 | 0·0004487 | 0·004783 | 0·0032244 | Yes | 2·4987013 |  |  |  |  |  |
| CHL36 | 51 | 2 |  |  | 0 |  | 23 | 0·0015888 | 0·0007539 | 0·0059362 | 0·0045845 | Yes | 1·73454545 |  |  |  |  |  |
| CHL37 | 46 | 2 |  |  | 2 |  | 41 | 0·0010746 | 0·0005423 | 0·0048402 | 0·0030944 | Yes | 0·59272727 |  |  |  |  |  |
| CHL38 | 57 | 2 |  |  | 2 |  | 21 | 0·0012356 | 0·0010133 | 0·0072027 | 0·0065055 | Yes | 2·32727273 |  |  |  |  |  |
| CHL39 | 59 | 2 |  |  | 1 |  | 22 | 0·0012916 | 0·0004454 | 0·0043195 | 0·0031317 | Yes | 3·84207792 |  |  |  |  |  |
| CHL40 | 22 | 1 |  |  | 1 |  | 20 | 0·0019925 | 0·0006579 | 0·0057188 | 0·0048194 | Yes | 3·63480519 |  |  |  |  |  |
| CHL41 | 33 | 1 |  |  | 3 |  | 28 | 0·0026139 | 0·000628 | 0·0065606 | 0·0042356 | Yes | NR |  |  |  |  |  |
| CHL42 | 37 | 1 |  |  | 4 |  | 28 | 0·0016445 | 0·0005453 | 0·0042284 | 0·0028905 | Yes | NR |  |  |  |  |  |
| CHL43 | 47 | 2 |  |  | 0 |  | 28 | 0·003488 | 0·000581 | 0·0053692 | 0·0041611 | Yes | NR |  |  |  |  |  |
| CHL44 | 61 | 2 |  |  | 4 |  | 47 | 0·001475 | 0·0008775 | 0·0090757 | 0·0073949 | Yes | NR |  |  |  |  |  |
| CHL45 | 30 | 1 |  |  | 0 |  | 20 | 0·0018823 | 0·0008983 | 0·0071319 | 0·0058978 | NR | NR |  |  |  |  |  |
| CHL46 | 49 | 1 |  |  | 1 |  | 25 | 0·0013456 | 0·0005469 | 0·0041105 | 0·0032835 | NR | NR |  |  |  |  |  |
| CHL47 | 42 | 2 |  |  | 5 |  | 44 | 0·00195 | 0·0013218 | 0·0079622 | 0·0075158 | NR | NR |  |  |  |  |  |
| CHL48 | 27 | 1 |  |  | 0 |  | 26 | 0·0024104 | 0·0006113 | 0·0051486 | 0·0038232 | NR | NR |  |  |  |  |  |
| CHL49 | 57 | 2 |  |  | 0 |  | 24 | 0·0018089 | 0·0003736 | 0·0046135 | 0·0029769 | NR | NR |  |  |  |  |  |
| CHL50 | 25 | 1 |  |  | 2 |  | 35 | 0·0030502 | 0·0007919 | 0·0068183 | 0·0059494 | NR | NR |  |  |  |  |  |
| CHL51 | 36 | 2 |  |  | 3 |  | 29 | 0·0021217 | 0·0006858 | 0·0066818 | 0·0065761 | NR | NR |  |  |  |  |  |
| CHL52 | 37 | 1 |  |  | 1 |  | 34 | 0·0018123 | 0·000912 | 0·0083643 | 0·0088976 | NR | NR |  |  |  |  |  |
| CHL53 | 48 | 2 |  |  | 0 |  | 23 | 0·0016674 | 0·0004848 | 0·0058887 | 0·0035417 | NR | NR |  |  |  |  |  |
| CHL55 | 47 | 1 |  |  | 9 |  | 40 | 0·0023181 | 0·000623 | 0·0058533 | 0·004067 | NR | NR |  |  |  |  |  |
| CHL56 | 20 | 2 |  |  | 3 |  | 31 | 0·0015675 | 0·0010086 | 0·0069628 | 0·0065555 | NR | NR |  |  |  |  |  |
| CHL57 | 36 | 1 |  |  | 1 |  | 23 | 0·0037983 | 0·0006723 | 0·0067394 | 0·0039852 | NR | NR |  |  |  |  |  |
| CHL58 | 42 | 2 |  |  | 2 |  | 35 | 0·0011502 | 0·0004035 | 0·006082 | 0·0042415 | NR | NR |  |  |  |  |  |
| CHL59 | 42 | 2 |  |  | 0 |  | 21 | 0·0007156 | 0·0009968 | 0·0080693 | 0·0084761 | NR | NR |  |  |  |  |  |
| CHL60 | 25 | 1 |  |  | 0 |  | 20 | 0·0014444 | 0·0004395 | 0·0034139 | 0·0022462 | NR | NR |  |  |  |  |  |
| CHL62 | 23 | 2 |  |  | 5 |  | 45 | 0·0015881 | 0·0009532 | 0·0057963 | 0·0060635 | NR | NR |  |  |  |  |  |
| CHL63 | 35 | 2 |  |  | 1 |  | 21 | 0·0011781 | 0·0007161 | 0·0066856 | 0·0059716 | NR | NR |  |  |  |  |  |
| CHL64 | 38 | 1 |  |  | 0 |  | 25 | 0·0014884 | 0·000904 | 0·0065784 | 0·0068129 | NR | NR |  |  |  |  |  |
| CHL65 | 37 | 2 |  |  | 2 |  | 27 | 0·0016236 | 0·0004151 | 0·0067307 | 0·0050302 | NR | NR |  |  |  |  |  |

*^a^* The information if not provided in the questionnaire or not assayed in the experiments is marked as NR (not recorded); Pain duration and CPG scores were irrelevant for control samples

*^b^* Male and Female are coded as 1 and 2, respectively

*^c^* Samples used for gene expression are marked as yes

*^d^* Intake of anti-inflammatory, anticonvulsant and antidepressant drugs were recorded as binary variables under the column ANTIINF, ANTICONV and ANTIDEP, respectively. Drug if taken is marked as 1 or else 0.

**Table S2.** Clinical characteristics*^a^* of samples in cohort B; CNP and control samples codes start with NPG and CG, respectively.

| **CODE** | **AGE** | **GENDER**  **CODE*^b^*** | **DURATION**  **(months)** | **SLANSS** | **PHQ9** | **CPG** | **STAI-I** | **2-AG (pmol/μL)** | **AEA (pmol/μL)** | **PEA (pmol/μL)** | **OEA (pmol/μL)** | **GENE*^c^*** | **PC (mM)** | **ANTIINF*^d^*** | **ANTIDEP**^d^ | **ANTICONV*^d^*** | **OPIOIDS** |
| --- | --- | --- | --- | --- | --- | --- | --- | --- | --- | --- | --- | --- | --- | --- | --- | --- | --- |
| NPG001 | 49 | 2 | 240 | 18 | 11 | III | 52 | 0·005822 | 0·001607 | 0·011828 | 0·007382 | Yes | NR | 0 | 0 | 1 | 1 |
| NPG002 | 50 | 2 | 9 | 11 | 2 | II | 29 | 0·005689 | 0·000582 | 0·00565 | 0·003135 | Yes | NR | 0 | 1 | 0 | 0 |
| NPG003 | 63 | 1 | 72 | 24 | 7 | IV | 36 | 0·009122 | 0·001352 | 0·006824 | 0·00821 | Yes | NR | NR | NR | NR | NR |
| NPG004 | 72 | 2 | 240 | 13 | 10 | IV | 51 | 0·006534 | 0·002199 | 0·012523 | 0·015969 | Yes | NR | 0 | 0 | 1 | 0 |
| NPG005 | 68 | 2 | 36 | 16 | 12 | IV | 37 | 0·00399 | 0·002244 | 0·011057 | 0·016901 | Yes | NR | 1 | 1 | 0 | 1 |
| NPG007 | 72 | 2 | 72 | 13 | 10 | IV | 51 | 0·009307 | 0·001687 | 0·008497 | 0·009072 | Yes | NR | NR | NR | NR | NR |
| NPG008 | 44 | 2 | 48 | 16 | 14 | III | 52 | 0·012751 | 0·001755 | 0·010893 | 0·011159 | Yes | NR | 1 | 0 | 0 | 1 |
| NPG009 | 56 | 1 | 70 | 2 | 6 | III | 52 | 0·018734 | 0·001846 | 0·010552 | 0·008774 | Yes | 2·11688312 | 1 | 0 | 1 | 1 |
| NPG011 | 86 | 2 | 10 | 17 | 12 | IV | 46 | 0·019871 | 0·001474 | 0·006778 | 0·007056 | Yes | NR | 1 | 0 | 0 | 1 |
| NPG012 | 71 | 1 | 48 | 16 | 10 | III | 35 | 0·002118 | 0·001071 | 0·008977 | 0·009792 | Yes | NR | 0 | 1 | 1 | 1 |
| NPG013 | 47 | 2 | 120 | 24 | 22 | III | 49 | 0·008294 | 0·001616 | 0·011444 | 0·013497 | Yes | NR | 1 | 0 | 1 | 0 |
| NPG014 | 56 | 2 | 36 | 11 | 13 | IV | 43 | 0·018725 | 0·00177 | 0·008121 | 0·007095 | Yes | 2·87662338 | NR | NR | NR | NR |
| NPG015 | 54 | 2 | 24 | 7 | 6 | I | 43 | 0·005644 | 0·00164 | 0·009235 | 0·007078 | Yes | NR | NR | NR | NR | NR |
| NPG016 | 51 | 2 | 84 | 24 | 21 | IV | 66 | 0·0011 | 0·001167 | 0·012842 | 0·015609 | Yes | NR | 0 | 0 | 1 | 1 |
| NPG017 | 58 | 1 | 240 | 24 | 16 | IV | 52 | 0·005559 | 0·002029 | 0·011245 | 0·006043 | Yes | 3·43571429 | NR | NR | NR | NR |
| NPG019 | 28 | 1 | 36 | 2 | 7 | II | 36 | 0·005616 | 0·00194 | 0·00968 | 0·008994 | Yes | NR | 1 | 0 | 0 | 0 |
| NPG020 | 49 | 1 | 73 | 5 |  | NR | NR | 0·008015 | 0·00194 | 0·010591 | 0·008654 | Yes | NR | 1 | 0 | 0 | 0 |
| NPG021 | 63 | 1 | 120 | 9 | 7 | I | 37 | 0·002046 | 0·001294 | 0·007723 | 0·004736 | Yes | NR | NR | NR | NR | NR |
| NPG022 | 23 | 2 | 48 | 12 | 19 | III | 44 | 0·001212 | 0·001935 | 0·009767 | 0·01171 | Yes | NR | NR | NR | NR | NR |
| NPG023 | 58 | 1 | 16 | 8 | 0 | I | 34 | 0·010925 | 0·001765 | 0·01095 | 0·007618 | Yes | NR | NR | NR | NR | NR |
| NPG024 | 49 | 1 | 168 | 9 | 4 | III | 29 | 0·012464 | 0·002687 | 0·012312 | 0·013226 | Yes | NR | 1 | 0 | 1 | 0 |
| NPG025 | 36 | 2 | 96 | 16 | 3 | III | 39 | 0·006383 | 0·001527 | 0·007667 | 0·007654 | Yes | 4·63116883 | NR | NR | NR | NR |
| NPG026 | 48 | 1 | 240 | 6 | 5 | IV | 22 | 0·004122 | 0·001639 | 0·011185 | 0·006288 | Yes | 3·13051948 | 1 | 0 | 0 | 0 |
| NPG027 | 57 | 2 | 252 | 16 | 9 | III | 45 | 0·002349 | 0·002675 | 0·013615 | 0·014119 | Yes | NR | NR | NR | NR | NR |
| NPG028 | 40 | 1 | 60 | 8 | 0 | I | 27 | 0·013745 | 0·002122 | 0·009109 | 0·007688 | Yes | NR | NR | NR | NR | NR |
| NPG029 | 43 | 2 | 42 | 24 | 3 | III | 36 | 0·010496 | 0·001906 | 0·012424 | 0·010549 | Yes | NR | 0 | 1 | 1 | 0 |
| NPG030 | 42 | 1 | 24 | 5 | 5 | I | 44 | 0·020575 | 0·001189 | 0·009331 | 0·004585 | Yes | NR | 0 | 0 | 1 | 0 |
| NPG031 | 27 | 2 | 48 | 18 | 14 | IV | 57 | 0·003559 | 0·001836 | 0·00928 | 0·00843 | Yes | NR | NR | NR | NR | NR |
| NPG032 | 43 | 2 | 156 | 17 | 5 | III | 46 | 0·003942 | 0·001513 | 0·009667 | 0·010567 | Yes | NR | NR | NR | NR | NR |
| NPG033 | 54 | 1 | 48 | 11 | 2 | II | 45 | 0·003702 | 0·001315 | 0·006459 | 0·006463 | Yes | 2·43896104 | 1 | 0 | 0 | 0 |
| NPG034 | 44 | 1 | 23 | 24 | 19 | IV | 56 | 0·010395 | 0·001546 | 0·0083 | 0·007197 | Yes | NR | NR | NR | NR | NR |
| NPG035 | 72 | 2 | 36 | 3 | 1 | I | 31 | 0·004944 | 0·000698 | 0·005832 | 0·004067 | Yes | NR | 0 | 0 | 1 | 0 |
| NPG036 | 30 | 2 | 24 | 14 | 12 | IV | 34 | 0·002666 | 0·001615 | 0·006837 | 0·005931 | Yes | 3·31363636 | 0 | 0 | 1 | 0 |
| NPG037 | 41 | 2 | 12 | 18 | 19 | III | 43 | 0·005352 | 0·001836 | 0·011273 | 0·007726 | NR | NR | 0 | 1 | 1 | 0 |
| NPG038 | 48 | 2 | 36 | 8 | 5 | III | 42 | 0·017443 | 0·001586 | 0·008936 | 0·008063 | Yes | NR | 0 | 0 | 1 | 0 |
| NPG039 | 46 | 1 | 36 | 3 | 10 | III | 51 | 0·003097 | 0·001854 | 0·01028 | 0·012043 | Yes | 2·6474026 | 0 | 0 | 0 | 1 |
| NPG040 | 79 | 2 | 24 | 24 | 3 | III | 20 | 0·002153 | 0·001066 | 0·006711 | 0·006847 | Yes | 2·26818182 | 0 | 0 | 1 | 0 |
| NPG041 | NR | 2 | 24 | 0 | 7 | III | 25 | 0·00957 | 0·002493 | 0·01225 | 0·01451 | Yes | NR | 0 | 0 | 0 | 1 |
| NPG042 | 58 | 2 | 24 | 0 | NR | NR | 49 | 0·004921 | 0·002248 | 0·010716 | 0·014367 | Yes | NR | 0 | 0 | 0 | 1 |
| NPG043 | 53 | 1 | 36 | 14 | 18 | IV | 64 | 0·010473 | 0·001909 | 0·011528 | 0·009014 | Yes | 2·86883117 | 1 | 0 | 0 | 0 |
| NPG044 | 37 | 1 | 84 | 14 | 15 | IV | NR | 0·003645 | 0·002536 | 0·010772 | 0·010164 | Yes | NR | 0 | 0 | 0 | 0 |
| NPG045 | 50 | 1 | 18 | 10 | 5 | III | 26 | 0·004794 | 0·001969 | 0·009223 | 0·008833 | Yes | 4·29675325 | 1 | 0 | 0 | 0 |
| NPG046 | 37 | 2 | 36 | 16 | 1 | NR | 45 | 0·003097 | 0·002263 | 0·012114 | 0·009898 | Yes | NR | NR | NR | NR | NR |
| NPG047 | 46 | 2 | 24 | 6 | 4 | IV | 31 | 0·001389 | 0·001325 | 0·007547 | 0·006965 | Yes | NR | 0 | 0 | 0 | 0 |
| NPG048 | 54 | 1 | 108 | 5 | 2 | III | NR | 0·003588 | 0·001564 | 0·010761 | 0·007281 | Yes | NR | 0 | 0 | 0 | 0 |
| NPG049 | 41 | 1 | 36 | 0 | 4 | II | 33 | 0·004286 | 0·00177 | 0·010512 | 0·008228 | Yes | NR | 0 | 0 | 1 | 0 |
| NPG050 | 50 | 1 | 60 | 14 | 10 | IV | 44 | 0·019064 | 0·001615 | 0·008743 | 0·007217 | Yes | NR | 0 | 1 | 1 | 1 |
| NPG051 | 54 | 2 | 24 | 2 | 13 | IV | 43 | 0·027674 | 0·001664 | 0·011044 | 0·007902 | Yes | 4·27402597 | 0 | 0 | 0 | 1 |
| NPG052 | 42 | 2 | 24 | 12 | 27 | III | 49 | 0·022351 | 0·003919 | 0·024335 | 0·024999 | Yes | NR | 0 | 0 | 0 | 1 |
| NPG053 | 63 | 1 | 84 | 11 | 1 | II | 20 | 0·007703 | 0·001174 | 0·006037 | 0·005424 | Yes | 1·90909091 | 0 | 0 | 0 | 1 |
| NPG054 | NR | NR | 6 | NR | NR | NR | NR | 0·028143 | 0·001652 | 0·010348 | 0·007684 | Yes | NR | NR | NR | NR | NR |
| NPG055 | 59 | 2 | 36 | 18 | 14 | III | 54 | 0·051351 | 0·001343 | 0·009753 | 0·006387 | Yes | NR | 1 | 0 | 1 | 0 |
| NPG056 | 63 | 2 | 36 | 0 | 0 | NR | NR | NR | NR | NR | NR | Yes | NR | 0 | 0 | 0 | 0 |
| NPG057 | 64 | 2 | 324 | 18 | 12 | IV | 55 | 0·004607 | 0·001138 | 0·006757 | 0·005813 | Yes | NR | 1 | 1 | 1 | 1 |
| NPG058 | 39 | 1 | 36 | 13 | 7 | I | 44 | 0·00605 | 0·001996 | 0·010037 | 0·010834 | Yes | NR | 0 | 0 | 0 | 0 |
| NPG059 | 49 | 2 | 120 | 13 | NR | III | NR | 0·001332 | 0·001346 | 0·011652 | 0·008131 | Yes | NR | 0 | 0 | 0 | 1 |
| NPG060 | 50 | 2 | 60 | 5 | 13 | IV | 42 | NR | NR | NR | NR | Yes | 2·60974026 | 1 | 0 | 0 | 1 |
| NPG061 | NR | NR | 36 | NR | NR | NR | NR | 0·002399 | 0·002038 | 0·010295 | 0·010004 | Yes | NR | NR | NR | NR | NR |
| NPG062 | 49 | 1 | 240 | 8 | 12 | IV | 36 | 0·00519 | 0·001573 | 0·008059 | 0·007322 | Yes | NR | 0 | 0 | 0 | 1 |
| NPG063 | 63 | 1 | 60 | 13 | 6 | IV | 48 | 0·006932 | 0·001431 | 0·008397 | 0·005837 | Yes | 3·79350649 | 0 | 1 | 1 | 1 |
| NPG064 | 55 | 2 | 360 | 13 | 14 | II | 42 | 0·005968 | 0·001373 | 0·007178 | 0·006071 | Yes | NR | 0 | 0 | 1 | 0 |
| NPG065 | 56 | 1 | 24 | 18 | 11 | III | 29 | 0·007397 | 0·001243 | 0·006991 | 0·004469 | Yes | 3·27012987 | 0 | 0 | 0 | 0 |
| NPG067 | 58 | 1 | 24 | 0 | 1 | II | 22 | 0·011502 | 0·00179 | 0·008412 | 0·007329 | Yes | NR | 1 | 0 | 0 | 0 |
| NPG068 | 62 | 2 | 264 | 17 | 5 | II | 20 | 0·003186 | 0·000788 | 0·006907 | 0·005267 | Yes | NR | 0 | 0 | 0 | 1 |
| NPG069 | 43 | 2 | 36 | 11 | 5 | II | 27 | 0·006487 | 0·001339 | 0·010037 | 0·008944 | Yes | 4·91883117 | 1 | 0 | 0 | 1 |
| NPG070 | 47 | 1 | 180 | 19 | 23 | II | 63 | 0·014941 | 0·001247 | 0·006882 | 0·005628 | Yes | NR | 0 | 0 | 1 | 1 |
| NPG071 | 86 | 2 | 96 | 3 | 4 | IV | 36 | 0·005873 | 0·001457 | 0·006657 | 0·006639 | Yes | NR | 1 | 0 | 0 | 1 |
| NPG072 | NR | NR | 168 | 6 | 0 | II | 21 | 0·009557 | 0·002109 | 0·009389 | 0·010667 | Yes | NR | 1 | 0 | 1 | 0 |
| NPG073 | NR | NR | 180 | NR | NR | NR | NR | 0·007112 | 0·001339 | 0·010411 | 0·010457 | Yes | NR | NR | NR | NR | NR |
| NPG074 | 60 | 2 | 144 | 14 | 12 | III | 50 | 0·010727 | 0·001397 | 0·007862 | 0·006327 | Yes | NR | 1 | 0 | 1 | 0 |
| NPG075 | NR | 2 | 48 | 11 | 4 | II | 35 | 0·006331 | 0·001058 | 0·007303 | 0·004778 | Yes | NR | 1 | 0 | 0 | 0 |
| NPG076 | 50 | 1 | 18 | 6 | 5 | II | 34 | 0·006861 | 0·001154 | 0·007548 | 0·005617 | Yes | NR | 1 | 1 | 1 | 0 |
| NPG077 | 54 | 2 | 6 | 0 | 7 | II | 36 | 0·015233 | 0·0028 | 0·010702 | 0·014371 | Yes | NR | 1 | 0 | 0 | 0 |
| NPG078 | NR | 1 | 24 | NR | NR | III | 31 | 0·01259 | 0·001433 | 0·009413 | 0·007524 | Yes | NR | 0 | 0 | 1 | 1 |
| NPG079 | NR | NR | 6 | NR | NR | NR | NR | 0·019515 | 0·001804 | 0·010463 | 0·009152 | Yes | NR | NR | NR | NR | NR |
| NPG080 | NR | NR | NR | NR | NR | NR | NR | 0·022393 | 0·004142 | 0·013843 | 0·01955 | Yes | NR | NR | NR | NR | NR |
| NPG081 | NR | NR | 24 | NR | NR | NR | NR | 0·008109 | 0·001642 | 0·010765 | 0·009407 | Yes | NR | NR | NR | NR | NR |
| NPG082 | 61 | 1 | 36 | 23 | 17 | IV | 49 | 0·016708 | 0·002832 | 0·013398 | 0·011381 | Yes | NR | 1 | 0 | 0 | 0 |
| CG01 | NR | NR |  |  | NR |  | NR | 0·007956 | 0·001754 | 0·009649 | 0·011228 | Yes | NR |  |  |  |  |
| CG02 | 43 | 2 |  |  | 3 |  | 40 | 0·001602 | 0·001 | 0·007214 | 0·006072 | Yes | NR |  |  |  |  |
| CG03 | 60 | 1 |  |  | 0 |  | 46 | 0·004648 | 0·002099 | 0·017747 | 0·013158 | Yes | NR |  |  |  |  |
| CG05 | 61 | 2 |  |  | 3 |  | NR | 0·001906 | 0·001624 | 0·011278 | 0·00945 | Yes | 5·13506494 |  |  |  |  |
| CG07 | 30 | 2 |  |  | 1 |  | 34 | 0·002174 | 0·001812 | 0·009586 | 0·00921 | Yes | NR |  |  |  |  |
| CG08 | 44 | 2 |  |  | 0 |  | 31 | 0·001508 | 0·000549 | 0·005108 | 0·003914 | Yes | 5·70454545 |  |  |  |  |
| CG12 | 72 | 1 |  |  | 1 |  | 20 | 0·003864 | 0·001254 | 0·010088 | 0·006736 | Yes | 5·62012987 |  |  |  |  |
| CG13 | 49 | 2 |  |  | 0 |  | 20 | 0·004353 | 0·001031 | 0·007951 | 0·005894 | Yes | 4·12337662 |  |  |  |  |
| CG14 | 56 | 2 |  |  | 0 |  | 30 | 0·004629 | 0·002237 | 0·009979 | 0·010645 | Yes | 4·78051948 |  |  |  |  |
| CG15 | 54 | 2 |  |  | 1 |  | 28 | 0·002769 | 0·002169 | 0·011484 | 0·010481 | Yes | 5·1525974 |  |  |  |  |
| CG16 | 57 | 2 |  |  | 0 |  | 20 | 0·0064 | 0·002506 | 0·010614 | 0·010674 | Yes | 6·72012987 |  |  |  |  |
| CG17 | 58 | 1 |  |  | 0 |  | 20 | 0·00723 | 0·002326 | 0·01513 | 0·015564 | Yes | 3·25064935 |  |  |  |  |
| CG18 | 44 | 2 |  |  | 0 |  | 20 | 0·001432 | 0·001411 | 0·009174 | 0·009303 | Yes | NR |  |  |  |  |
| CG19 | 25 | 1 |  |  | 8 |  | 36 | 0·00492 | 0·00124 | 0·006964 | 0·005227 | Yes | NR |  |  |  |  |
| CG20 | 47 | 1 |  |  | 0 |  | 20 | 0·007144 | 0·001717 | 0·009552 | 0·008722 | Yes | NR |  |  |  |  |
| CG24 | 40 | 1 |  |  | 5 |  | 46 | 0·003926 | 0·001059 | 0·008013 | 0·006764 | Yes | NR |  |  |  |  |
| CG25 | 36 | 2 |  |  | 2 |  | 43 | 0·001992 | 0·001051 | 0·005144 | 0·004811 | Yes | NR |  |  |  |  |
| CG26 | 36 | 1 |  |  | 1 |  | 23 |  |  |  |  | Yes | NR |  |  |  |  |
| CG27 | 57 | 2 |  |  | 0 |  | 29 | 0·007296 | 0·001506 | 0·009677 | 0·008029 | Yes | NR |  |  |  |  |
| CG28 | 39 | 1 |  |  | 0 |  | 26 | 0·003382 | 0·003761 | 0·014768 | 0·017393 | Yes | NR |  |  |  |  |
| CG29 | 34 | 2 |  |  | 0 |  | 27 |  |  |  |  | Yes | NR |  |  |  |  |
| CG30 | 42 | 1 |  |  | 0 |  | 20 | 0·005542 | 0·001961 | 0·009475 | 0·008419 | Yes | NR |  |  |  |  |
| CG31 | 27 | 2 |  |  | 0 |  | 26 | 0·001369 | 0·00061 | 0·003596 | 0·002683 | Yes | 3·29805195 |  |  |  |  |
| CG32 | 45 | 2 |  |  | 1 |  | 28 |  |  |  |  | Yes | NR |  |  |  |  |
| CG34 | 42 | 1 |  |  | 0 |  | 28 | 0·001474 | 0·001998 | 0·011855 | 0·010596 | Yes | NR |  |  |  |  |
| CG35 | 24 | 2 |  |  | 2 |  | 41 | 0·002805 | 0·001489 | 0·007189 | 0·008989 | Yes | NR |  |  |  |  |
| CG36 | 36 | 2 |  |  | 5 |  | 28 | 0·001613 | 0·001065 | 0·00768 | 0·006619 | Yes | NR |  |  |  |  |
| CG37 | 29 | 2 |  |  | 1 |  | 26 | 0·003696 | 0·00084 | 0·00569 | 0·004502 | Yes | NR |  |  |  |  |
| CG38 | 40 | 2 |  |  | 1 |  | 24 | 0·001744 | 0·00125 | 0·008314 | 0·005996 | Yes | 4·17597403 |  |  |  |  |
| CG39 | 29 | 1 |  |  | 0 |  | 24 | 0·005562 | 0·001312 | 0·007817 | 0·006307 | Yes | 5·33311688 |  |  |  |  |
| CG40 | 69 | 2 |  |  | 0 |  | NR | 0·001518 | 0·001903 | 0·012065 | 0·008119 | Yes | 4·67337662 |  |  |  |  |
| CG41 |  |  |  |  |  |  | NR | 0·001313 | 0·000987 | 0·005656 | 0·004609 | Yes | NR |  |  |  |  |
| CG42 | 59 | 2 |  |  | 0 |  | 21 | 0·005291 | 0·00273 | 0·014196 | 0·012158 | Yes | NR |  |  |  |  |
| CG43 |  |  |  |  |  |  | NR | 0·003924 | 0·003523 | 0·015275 | 0·01665 | Yes | NR |  |  |  |  |
| CG44 | 35 | 1 |  |  | 0 |  | 20 | 0·001801 | 0·000865 | 0·007041 | 0·00576 | Yes | NR |  |  |  |  |
| CG45 | 51 | 1 |  |  | 0 |  | 23 | 0·006958 | 0·001213 | 0·00732 | 0·006027 | Yes | 5·30064935 |  |  |  |  |
| CG46 | 37 | 2 |  |  | 12 |  | 42 | 0·006847 | 0·002231 | 0·008583 | 0·008079 | Yes | NR |  |  |  |  |
| CG47 | 45 | 2 |  |  | 0 |  | 21 |  |  |  |  | Yes | NR |  |  |  |  |
| CG48 | 56 | 1 |  |  | 0 |  | 20 | 0·007184 | 0·00116 | 0·007048 | 0·007045 | Yes | 5·750000000 |  |  |  |  |
| CG49 | 39 | 1 |  |  | 0 |  | ? |  |  |  |  | Yes | NR |  |  |  |  |
| CG50 | 52 | 1 |  |  | 0 |  | 25 | 0·003144 | 0·002562 | 0·012483 | 0·011298 | Yes | NR |  |  |  |  |
| CG51 | 56 | 2 |  |  | 0 |  | 20 | 0·005867 | 0·001322 | 0·008249 | 0·00708 | Yes | NR |  |  |  |  |
| CG52 | 31 | 2 |  |  | 7 |  | 38 |  |  |  |  | Yes | 7·75779221 |  |  |  |  |
| CG53 | 68 | 1 |  |  | 0 |  | 20 | 0·00968 | 0·001017 | 0·007676 | 0·005857 | Yes | NR |  |  |  |  |
| CG54 | 57 | 1 |  |  | 1 |  | 23 | 0·004319 | 0·002368 | 0·011251 | 0·012085 | Yes | NR |  |  |  |  |
| CG55 | 71 | 2 |  |  | 0 |  | 20 | 0·001897 | 0·000467 | 0·004604 | 0·003181 | Yes | 5·46623377 |  |  |  |  |
| CG56 | 63 | 2 |  |  | 1 |  | 48 | 0·005292 | 0·003593 | 0·015357 | 0·018439 | Yes | NR |  |  |  |  |
| CG57 | 56 | 2 |  |  | 1 |  | 20 | 0·003644 | 0·001174 | 0·008626 | 0·008076 | Yes | 5·76038961 |  |  |  |  |
| CG59 | 42 | 2 |  |  | 0 |  | 40 |  |  |  |  | Yes | NR |  |  |  |  |
| CG60 | 51 | 2 |  |  | 0 |  | 35 | 0·001244 | 0·001791 | 0·009933 | 0·009793 | Yes | NR |  |  |  |  |
| CG61 | 40 | 2 |  |  | 2 |  | 30 | 0·003682 | 0·000746 | 0·006048 | 0·004234 | Yes | NR |  |  |  |  |
| CG63 | 65 | 1 |  |  | 1 |  | 22 | 0·006434 | 0·001383 | 0·007532 | 0·006924 | Yes | NR |  |  |  |  |
| CG64 | 57 | 2 |  |  | 2 |  | 35 | 0·012977 | 0·001175 | 0·00721 | 0·006003 | Yes | NR |  |  |  |  |
| CG66 | 52 | 2 |  |  | 0 |  | 44 | 0·005784 | 0·001369 | 0·008753 | 0·008976 | Yes | 5·16168831 |  |  |  |  |
| CG67 | 78 | 1 |  |  | 1 |  | 21 | 0·010534 | 0·001246 | 0·007985 | 0·005287 | Yes | NR |  |  |  |  |
| CG68 | 52 | 2 |  |  | 2 |  | 25 | 0·002378 | 0·002632 | 0·011535 | 0·014606 | Yes | NR |  |  |  |  |
| CG70 | 48 | 1 |  |  | 3 |  | 32 | 0·006011 | 0·002301 | 0·010718 | 0·013384 | Yes | 4·63116883 |  |  |  |  |
| CG72 | 74 | 1 |  |  | 0 |  | 20 |  |  |  |  | Yes | NR |  |  |  |  |
| CG73 | 57 | 2 |  |  | 0 |  | 25 | 0·003025 | 0·001653 | 0·010139 | 0·008945 | Yes | 6·3461039 |  |  |  |  |
| CG74 | 62 | 2 |  |  | 0 |  | 31 | 0·001776 | 0·000655 | 0·005533 | 0·003363 | Yes | NR |  |  |  |  |
| CG76 | 53 | 2 |  |  | 2 |  | 32 | 0·001675 | 0·000868 | 0·008763 | 0·006785 | Yes | NR |  |  |  |  |
| CG77 | 24 | 2 |  |  | 0 |  | 38 | 0·001652 | 0·001128 | 0·006326 | 0·006064 | Yes | NR |  |  |  |  |
| CG79 | 74 | 2 |  |  | 1 |  | 38 | 0·002264 | 0·000504 | 0·004548 | 0·003206 | NR | NR |  |  |  |  |
| CG80 | 71 | 1 |  |  | 2 |  | 20 | 0·004076 | 0·000914 | 0·006976 | 0·005123 | Yes | NR |  |  |  |  |
| CG100 | 74 | 1 |  |  | 9 |  | 38 | 0·013082 | 0·001378 | 0·007943 | 0·006788 | Yes | NR |  |  |  |  |

*^a^* The information if not provided in the questionnaire or not assayed in the experiments is marked as NR (not recorded); Pain duration and CPG scores were irrelevant for control samples

*^b^* Male and Female are coded as 1 and 2, respectively

*^c^* Samples used for gene expression are marked as yes

^d^ Intake of anti-inflammatory, anticonvulsant and antidepressant drugs were recorded as binary variables under the column ANTIINF, ANTICONV and ANTIDEP, respectively. Drug if taken is marked as 1 or else 0.

**Table S3.** Marginal means and associated parameters for analysis of the effect of CNP/control group on the gene expression in the combined cohorts.

| **Gene** | **Group** | **Marginal mean*** | **95% CI for marginal mean*** | **Bonferroni-corrected p-value** |
| --- | --- | --- | --- | --- |
| *CHPT1* | Control | 3·47 | (3·24, 3·71) | <0·001 |
|  | CNP | 2·78 | (2·60, 2·96) |  |
| *FAAH* | Control | 6·09 | (5·81, 6·06) | 0·517 |
|  | CNP | 6·26 | (6·01, 6·50) |  |
| *CNR1* | Control | 17·0 | (16·7, 17·3) | 0·590 |
|  | CNP | 16·9 | (16·7, 17·2) |  |
| *DAGLA* | Control | 10·4 | (10·1, 10·7) | 0·400 |
|  | CNP | 10·3 | (10·0, 10·5) |  |
| *MGLL* | Control | 5·28 | (5·08, 5·48) | 0·001 |
|  | CNP | 5·71 | (5·53, 5·88) |  |
| *NAAA* | Control | 6·31 | (6·11, 6·51) | 0·486 |
|  | CNP | 6·35 | (6·17, 6·53) |  |
| *NAPEPLD* | Control | 10·7 | (10·5, 10·9) | 0·012 |
|  | CNP | 10·9 | (10·8, 11·0) |  |

*Based on ∆Ct values. Note that ∆Ct values are inversely proportional to the gene expression.
